# MXgap: A MXene Learning Tool for Bandgap Prediction

**DOI:** 10.1021/acscatal.5c04191

**Published:** 2025-08-05

**Authors:** Diego Ontiveros, Sergi Vela, Francesc Viñes, Carmen Sousa

**Affiliations:** † Departament de Ciència de Materials i Química Física & Institut de Química Teòrica i Computacional (IQTCUB), 16724 Universitat de Barcelona, c/Martí i Franquès 1-11, 08028 Barcelona, Spain; ‡ Institut de Química Avançada de Catalunya (IQAC−CSIC), Barcelona 08034, Spain

**Keywords:** MXenes, machine learning, water splitting, density functional theory, photocatalysis

## Abstract

The increasing demand for clean and renewable energy has intensified the exploration of advanced materials for efficient photocatalysis, particularly for water splitting applications. Among these materials, MXenes, a family of two-dimensional (2D) transition metal carbides and nitrides, have shown great promise. This study leverages machine learning (ML) to address the resource-intensive process of predicting the bandgap of MXenes, which is critical for their photocatalytic performance. Using an extensive data set of 4356 MXene structures, we trained multiple ML models and developed a robust classifier-regressor pipeline that achieves a classification accuracy of 92% and a mean absolute error (MAE) of 0.17 eV for bandgap prediction. This framework, implemented in an open-source Python package, MXgap, has been applied to screen 396 La-based MXenes, identifying six promising candidates with suitable band alignments for water splitting whose optical properties were further explored via optical absorption and solar-to-hydrogen (STH) efficiency. These findings demonstrate the potential of ML to accelerate MXene discovery and optimization for energy applications.

## Graphical abstract

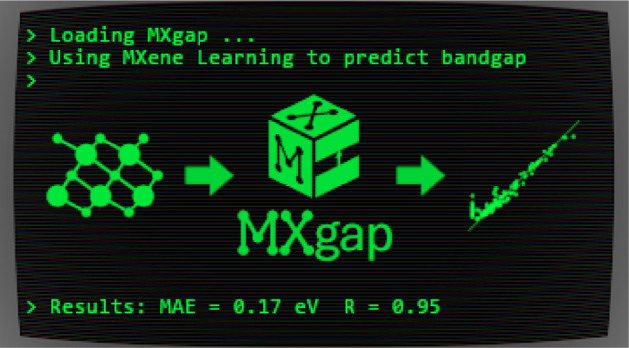

## 1 Introduction

As global energy demands continue to rise, the quest for clean, sustainable, and efficient energy sources has become increasingly urgent, driving extensive research into advanced materials for energy conversion and storage applications.^1^ Among these applications, photocatalysis has emerged as a critical process, enabling the direct conversion of solar energy —a renewable and widely available energy resource— into chemical energy to trigger reactions that can produce clean fuels and/or mitigate greenhouse gases.^2,3^ One of the most notable photocatalytic reactions is water splitting, in which sunlight is used to decompose water (H_2_O) into hydrogen (H_2_) and oxygen (O_2_). H_2_ generated via this process serves as a clean and efficient energy carrier that can be used as a potential carbon-free fuel.^4^ The water splitting reaction relies on the availability of H_2_O, an abundant and renewable resource, making it a highly sustainable process with significant potential for large-scale adoption. Beyond water splitting, photocatalysis is also gaining traction for other essential reactions, such as carbon dioxide (CO_2_) reduction, where photocatalysis can help reduce atmospheric CO_2_, a primary greenhouse gas, by converting it into valuable chemicals and fuels like methane, methanol, and carbon monoxide.^5^ However, efficient photocatalytic reactions require materials that can effectively absorb visible sunlight and facilitate the necessary redox reactions, with specific electronic properties such as an optimal bandgap for solar light absorption.^6^

Here, we focus on MXenes,^7^ a broad family of few-layered two-dimensional (2D) materials that have shown significant promise as photoactive materials. These are transition metal (TM) carbides and nitrides with M_*n*+1_X_*n*_ chemical formula, where M stands for an early TM from groups III to VI —*i.e.* Sc, Y, Ti, Zr, Hf, V, Nb, Ta, Cr, Mo, and W—, X can be carbon or nitrogen, and *n* = 1–4.^8–10^ Moreover, MXenes can have their surface easily functionalized with a termination, T_*x*_, thus updating the general chemical formula to M_*n*+1_X_*n*_T_*x*_. The usual synthesis of MXenes involves selectively etching A elements from bulk layered MAX materials precursors, M_*n*+1_AX_*n*_, where A is typically a *p*-group element.^11^ The etching process is commonly carried out using hydrofluoric acid (HF),^12^ which produces terminations such as −O, −F, −OH, and −H.^13^ Nevertheless, recent studies employing molten salts reported new MXenes terminated with −S, −Se, −Te, −NH, −Cl, −Br, and −I, resulting in a large family that encompasses thousands of compounds.^14,15^ MXenes are known for their tunable electronic properties, large surface area, and structural stability.^16^ These unique attributes have positioned MXenes at the front of global research,^17^ with applications spanning energy storage and electronics, where they excel in supercapacitors and batteries,^18^ to environmental applications such as water purification^19^ and CO_2_ capture and utilization.^20^ The ability to tailor the electronic structure of MXenes *via* their structure, composition, or surface termination unlocks exciting possibilities for enhancing their photocatalytic performance.^21,22^

A key feature in this context is the material bandgap, *E*_g_, directly affecting its ability to absorb sunlight and drive photochemical reactions.^6^ However, determining the bandgap and other properties often relies on resource-intensive approaches, such as direct experimental measurements or computational estimates employing density functional theory (DFT) simulations using hybrid functionals or advanced methods like those based on Green functions and screened Coulomb interactions (GW). Although these methods are highly accurate, they can become prohibitively expensive and time-consuming when applied across the vast design space of possible MXene compositions and structures, especially as this family of compounds continues to expand.^23^ There machine learning (ML) can be a transformative tool in materials science, leveraging vast data sets to identify patterns and make fast predictions.^24^ ML methods have already been successfully applied to predict a wide range of properties at different scales, including potential energies, crystal structures, electronic conductivities, and thermal stabilities.^25^ They are also making significant impacts across diverse fields, such as catalysis, surface science, environmental chemistry, biomaterials, and many others.^26,27^ In the context of MXenes, ML has also been applied to explore their thermodynamic stability,^28^ identifying new stable MXenes^29^ and predicting electronic properties such as work functions and HER catalytic activities.^30,31^ However, ML applications for MXenes electronic properties are still limited, with only few studies dedicated to predict their bandgaps or their bands alignments.^32,33^ Moreover, these existing studies primarily relied on traditional ML models, not providing readily accessible tools for further research or practical applications.

Here, we developed a new ML tool to predict the bandgap of MXene compounds, aiming to accelerate the discovery of MXenes that can act as efficient photocatalysts, illustrated here with water splitting, although of potential use in any phototriggered process. By using our previously published data on MXene electronic properties,^21,22^ we trained several ML models and developed an optimal model combining a ML classifier with a ML regressor. This model classifies each MXene as metallic or semiconducting with 92% accuracy and predicts the bandgap with a mean absolute error (MAE) of 0.17 eV. Unlike previous studies, our work systematically evaluates a broad range of models, including advanced tree ensemble methods, leading to improved predictive performance. Furthermore, we ensure that our results are also widely accessible by integrating these models into MXgap, a free and open-source Python package. Finally, to validate the tool, we screened 396 novel La-based MXenes and successfully identified six candidates with suitable band alignment for water splitting applications. These candidates were further validated by PBE0 computations and examined by optical absorption spectra and solar-to-hydrogen (STH) efficiency. The developed ML tool not only allows for discovering new photoactive MXenes but also for rapidly screening other bandgap-dependent properties of MXenes, such as those relevant to solar cells and photovoltaic applications.^34^

## 2 Methods

### 2.1 Structural Models

Pristine MXene structures, with the M_*n*+1_X_*n*_ chemical formula, consist of intercalated close-packed layers of M and X atoms, with the number of layers determined by the value of *n*. Two stacking arrangements are possible: ABC stacking, with the M layers in two different relative positions, or ABA stacking, with the M layers aligned in the same position along the vacuum direction; see Figure 1b. For terminated MXenes, M_*n*+1_X_*n*_T_2_, the T atoms can be added into different surface hollow sites of the pristine MXene structures, as indicated in Figure 1c. The explored sites are the metal hollow in ABC stacking, H_M_, located above an underlying metal atom, the simple hollow in ABA stacking, H, placed with no atoms underneath, the carbon or nitrogen hollows, H_X_, with an underlying X atom for both stackings, and a mixture of H_M_ (H) and H_X_ on opposite MXene surfaces for ABC (ABA) stacking, H_MX_. Combining stacking and hollow sites yields six possible configurations for each terminated MXene. These structures were modeled using a *p*(1×1) hexagonal unit cell, represented as a slab model with 30 Å of vacuum perpendicular to the MXene 2D surface. The structures used in this study are derived from a previous high-throughput computational screening aimed at assessing MXenes for photocatalytic water splitting.^21,22^

**Figure 1:**
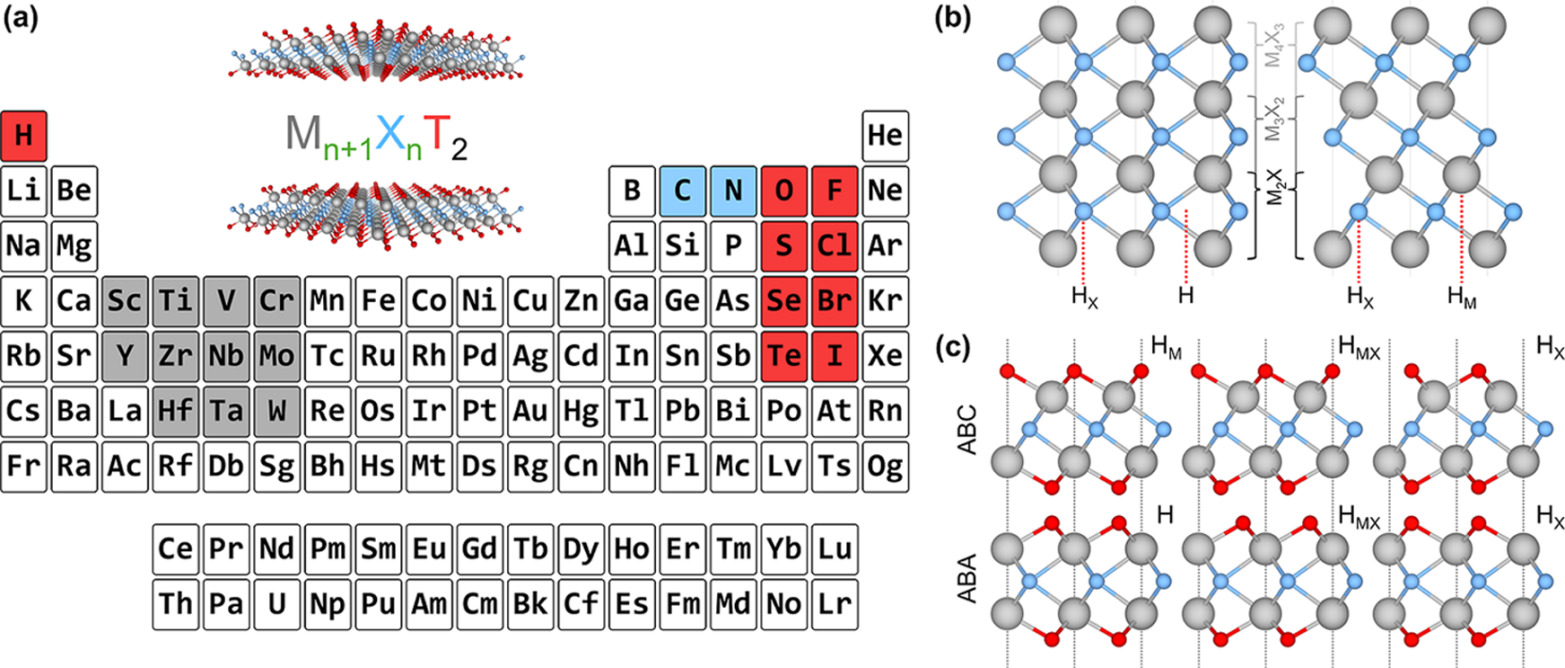
MXene composition and structure. (a) Periodic table showing the building blocks of MXenes, with the M, X, and T atoms colored in gray, blue, and red, respectively. (b) Side view of the ABA (left) and ABC (right) stackings for pristine MXenes. (c) Side views of the six possible configurations for a terminated MXene, depending on the stacking (ABC or ABA) and the termination position (H_M_/H, H_MX_, or H_X_).

### 2.2 Data set and Model Training

The data set used for model training is a collection of the structural and electronic properties of 4356 different MXene structures, considering the geometries and compositions represented in Figure 1a —*i.e.* M = Sc, Y, Ti, Zr, Hf, V, Nb, Ta, Cr, Mo, or W; X = C or N; T = F, Cl, Br, I, O, S, Se, Te, H, OH, NH; *n* = 1–3; and six geometries for each MXene. These selected compositions are based on those present in already synthesized MXenes.^15,35^ Each structure was characterized by a set of elemental, structural, and electronic features, detailed in Table S1 of the Supporting Information (SI). For model selection, a 60/20/20 train/validation/test split strategy was employed, using MAE and *R* metrics for performance evaluation in the regression tasks and the accuracy, precision, recall, and receiver operating characteristic (ROC) curve for classification. More information about the training and evaluation metrics can be found in the corresponding Section S1 and Figure S1 of the SI. During the training phase, data was randomly divided, reserving the test set for the final evaluation. A five-fold cross-validated grid search was then conducted to determine the optimal hyperparameters for each model. The best set of hyperparameters —*i.e.*, those that showed the highest validation score— were then used to train the final model, with the full training set, which was subsequently evaluated on the test set to ensure robust predictive performance. Six different ML algorithms were trained with the MXene data: Gradient boosting (GB), random forest (RF), support vector machine (SV), and multilayer perceptron (MLP) classifiers and regressors, plus logistic regression (LR) and kernel ridge regressor (KRR). Thus, the GB, RF, SV, and MLP models were trained for both classification of MXenes into metallic or semiconductor and regression to predict the bandgap, while LR was exclusively used for classification, as it cannot handle regression tasks, whereas KRR was used only for bandgap prediction. These ML algorithms are executed using the extensively adopted open-source Scikit-Learn library^36^ and are further explained in Section S2 of the SI.

### 2.3 DFT Methods

The electronic structure of the MXenes studied was analyzed within the framework of DFT,^37,38^ with calculations conducted through the Vienna ab initio simulation package (VASP).^39^ In these calculations, core electrons and their interactions with valence electrons were represented by projector augmented wave (PAW) pseudopotentials,^40^ and valence electrons were described with a plane-wave basis set, employing a 415 eV kinetic energy cutoff and considering spin-polarization. The Perdew–Burke–Ernzerhof (PBE)^41^ exchange–correlation functional was utilized under the generalized gradient approximation (GGA).^42^ Additionally, for a more accurate estimation of the bandgap and electronic structure, calculations incorporating the PBE0 hybrid functional,^43^ which includes 25% nonlocal Fock exchange, were carried out. Geometry optimizations were considered converged when the forces on nuclei were below 0.01 eV·Å^–1^, with a 10^–6^ eV threshold set for electronic convergence. During optimization, atomic positions and cell parameters were allowed to relax. For Brillouin zone integration, optimal **Γ**-centered 7×7×1 Monkhorst–Pack **k**-point grids were applied.^44^

To study the ability to harness sunlight of the promising cases resulting from the ML screening, the absorption coefficient α­(ω) was computed and estimated with the following formula

1 $$\alpha \left(\omega \right)=\frac{\sqrt{2}\omega }{c}{\left[\sqrt{\varepsilon_{r}^{2}\left(\omega \right)+\varepsilon_{i}^{2}\left(\omega \right)}-\varepsilon_{r}\left(\omega \right)\right]}^{1/2}$$

where *c* is the speed of light, and ε_*r*_ and ε_*i*_ are the real and imaginary parts of the dielectric function, respectively. To accurately compute the dielectric function and the optical absorption spectra, the GW-BSE approach was employed, which combines the many-body perturbation Green’s function and screened Coulombic interaction (GW)^45^ with the Bethe–Salpeter Equation (BSE),^46^ which accounts for electron–electron and electron–hole interactions. An optimal plane-wave energy cutoff of 200 eV was found, together with 960 bands for the GW calculations, while eight occupied and 16 unoccupied bands were selected to describe the excitons in the BSE calculations. All the calculations were performed using a converged 13×13×1 Monkhorst–Pack **k**-point grid.

The STH efficiency has also been estimated, which is a key parameter for evaluating the efficiency of converting solar light into hydrogen fuel. Here, one computes the upper limit of the STH, as based on a previous work,^47^ assuming 100% efficiency of the catalytic reactions. The STH can be decomposed into light absorption, η_abs_, and carrier utilization, η_cu_, efficiencies, which take the form of the following equations

2 $$\eta_{abs}=\frac{\int_{E_{opt}}^{\infty }P\left(\omega \right)d\omega }{\int_{0}^{\infty }P\left(\omega \right)d\omega }$$

3 $$\eta_{cu}=\Delta G\frac{\int_{E}^{\infty }\frac{P\left(\omega \right)}{\omega }d\omega }{\int_{E_{opt}}^{\infty }P\left(\omega \right)d\omega }$$

where *P*(ω)­is the air mass at 1.5 atm thickness global (AM1.5G) solar energy flux at photon energy,^48^ Δ*G* is the water redox potential difference of 1.23 eV, and *E* is the photon energy that can be actually utilized for water splitting. Considering the existing barriers for the hydrogen evolution reaction (HER) and oxygen evolution reaction (OER), extra energy is demanded to overcome those barriers, which should be added in *E*. According to the previous reports, considering the overpotentials of OER and HER cocatalysts and the energy loss during carriers migration between materials, suitable overpotentials of 0.2 and 0.6 V are assumed for the HER and OER, respectively.^47,49,50^ Thus, the *E* value can be expressed as

4 $$E=\{\begin{array}{l} E_{opt}\\ E_{opt}+0.2-\chi_{H_{2}}\\ E_{opt}+0.6-\chi_{O_{2}}\\ E_{opt}+0.8-\chi_{H_{2}}-\chi_{O_{2}} \end{array}\begin{array}{l} \text{if}\;\chi_{H_{2}}\geq 0.2\;\text{and}{\;\chi }_{O_{2}}\geq 0.6\\ \text{if}\;\chi_{H_{2}}<0.2\;\text{and}\;\chi_{O_{2}}\geq 0.6\\ \text{if}\;\chi_{H_{2}}\geq 0.2\;\text{and}{\;\chi }_{O_{2}}<0.6\\ \text{if}{\;\chi }_{H_{2}}<0.2\;\text{and}{\;\chi }_{O_{2}}<0.6 \end{array}$$

where χ_H_2__and χ_O_2__are the overpotentials for the HER and OER, respectively, computed as the difference between the band edge (the valence band maximum, VBM, or the conduction band minimum, CBM) and the corresponding redox half reaction potential. With both of these contributions, the STH efficiency is defined as

5 $$\eta_{STH}=\eta_{abs}\cdot \eta_{cu}$$

For Janus structures, with a difference between vacuum levels of the two surfaces, Δϕ, like for S-, Se-, and Te-terminated MXenes, the intrinsic electric field does positive work for the separation of photon excited electrons and holes during the processing of photocatalytic water splitting, and therefore, it should be added into the total energy. Hence, the corrected STH efficiency of photocatalytic water splitting for 2D materials with intrinsic electric field, η_STH_^′^, is defined as

6 $$\eta_{STH}^{\prime }=\eta_{STH}\frac{\int_{0}^{\infty }P\left(\omega \right)d\omega }{\int_{0}^{\infty }P\left(\omega \right)d\omega +\Delta \phi \int_{E_{opt}}^{\infty }\frac{P\left(\omega \right)}{\omega }d\omega }$$

## 3 Results and Discussion

The primary goal here is to efficiently predict the semiconducting properties of MXenes with minimal effort using ML models while incorporating essential physical insights. Thus, at the beginning, only periodic table values —*i.e.* atomic numbers, electronegativity, atomic radius, *etc.*— and structural information extracted from PBE periodic optimization calculations —*i.e.* lattice parameter and MXene widths, bonds distances, termination adsorption sites, *etc.*— were used, hereafter referred to as elemental features. Nonetheless, to improve the predictions, supplementary features gained from PBE density of states (DOS) were added —mainly the PBE bandgap, bandgap edges, and averaged DOS— hereafter referred to as DOS features. After performing a feature selection *via* evaluating the feature importance based on the random forest regression (RFR) model, 33 elemental features and 103 DOS features were kept, which are found in Table S1 of the SI. Thus, the database comprises these selected features alongside the target property, the PBE0 bandgap, which provides a more accurate approximation of the real bandgap, for a total of 4356 MXene structures. From this database, the model training and testing followed the workflow shown in Figure 2. For more comprehensive details on the database and training procedures, refer to the corresponding Methods section.

**Figure 2:**
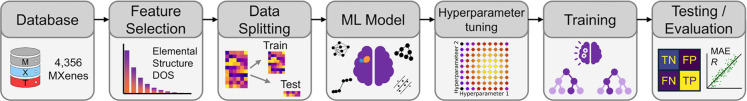
Machine learning workflow. From a database comprised of 4356 terminated MXenes, a set of features are selected to describe the MXenes. The data is split into a train set, used to train the ML model after the optimal hyperparameters are selected, and a test set, used to evaluate the model performance.

### 3.1 Bandgap Prediction with the Full Database

First, the full database was used, containing both metallic and semiconductor MXenes, in order to directly predict the bandgap of MXene structures. To choose the suitable ML algorithm, several models were evaluated for the regression, including gradient boosting regressor (GBR), random forest regressor (RFR), support vector regressor (SVR), multilayer perceptron regressor (MLPR), and KRR. These algorithms were chosen for their strengths in regression tasks and their ability to capture complex, nonlinear relationships, anticipated to be beneficial given the structural diversity in MXenes. Additionally, these models have been successfully applied in previous MXene compounds and bandgap prediction studies.^31,32,51^

After optimizing the hyperparameters and training the models using MXenes characterized solely by the elemental features, the models demonstrated the ability to predict the target property, the PBE0 bandgap, with MAE as low as 0.17 eV. The RFR model yielded the best results, as detailed in Table S2 of the SI. The correlation plots between the predicted and actual PBE0 bandgaps, presented in Figure S2 of the SI, indicate a reasonable agreement for the testing data, with a correlation coefficient, *R*, of around 0.84 across all models, with the exception of SVR with an *R* = 0.77. Despite having reasonable MAE and *R* metrics, this approach shows significant dispersion in the correlation plots and misclassifies many metallic MXenes as semiconductors (*cf.* Figure S2 of the SI). To improve the current predictions, the models were retrained including DOS features, as aforementioned. This results in a slight reduction in the MAE, down to 0.10 eV in the case of RFR, along with improvements in the correlation coefficient across all models, with the dispersion and the number of cases with errors exceeding 1 eV (*N*_ε>1_) decreasing significantly, as shown again in Table S2 and Figure S2 of the SI. Notably, ensemble forest-based models, that combine multiple decision trees, such as RFR and GBR, outperform other methods, including kernel-based approaches like KRR and SVR, as well as neural network models like MLPR. The latter models still exhibited substantial limitations, such as underestimating bandgaps for high-bandgap cases and showing high dispersion for metallic or low-bandgap MXenes, even predicting negative bandgap values in some cases, leading to persistently poor correlations. Therefore, the best-performing models so far are GBR and RFR trained with DOS features (see RFR results in Figure 3a).

**Figure 3:**
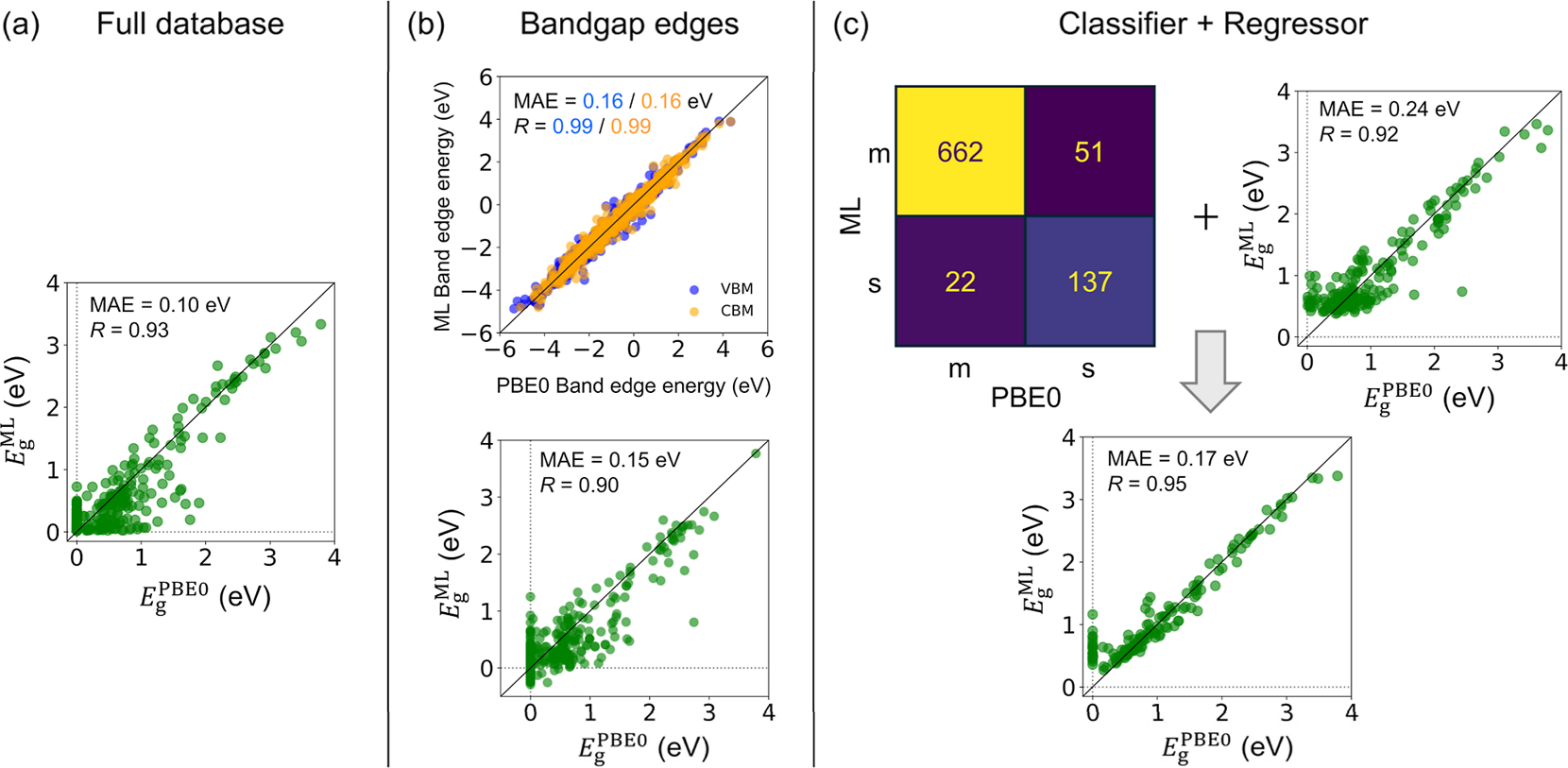
Approaches for bandgap prediction. (a) RFR prediction using the full database, including all metallic and semiconductor MXenes, showing the ML predicted bandgap, *E*_g_^ML^, *vs.* the PBE0 bandgap, *E*_g_^PBE0^, both shown in eV. (b) GBR bandgap prediction by separately estimating the VBM and CBM (top) and extracting the bandgap as the difference between the band edges. (c) A two-step approach: first, the GBC groups MXenes as either metallic (m) or semiconductor (s). For those predicted as semiconductors, a separate regression model is used to estimate their bandgap. The resulting correlation plot from combining both steps is shown at the bottom. Results in all cases are based on the test data set evaluation.

### 3.2 Bandgap Derived from Band Edges Prediction

The challenge in predicting the bandgap stems from the large number of metallic systems that share the same target value, despite variations in their input features. These cases of degeneracy make it difficult for the model to learn a univocal features-to-property map and, ultimately, to accurately predict the bandgap across all MXene cases, which leads to some metallic systems being misclassified as semiconductors. To tackle this issue, separate models were developed to predict the VBM and CBM at the PBE0 level without vacuum corrections. These models demonstrated strong predictive performance for each separate band edge, particularly when trained with DOS features, as shown in Table S3 and Figure S3 of the SI. Tree-based models once again achieved the best results, with MAEs of 0.16 and 0.18 eV for GBR and RFR, respectively, for both VBM and CBM energy predictions, and *R* ranging between 0.98 and 0.99. The GBR results are shown in Figure 3b. Nonetheless, when combining the predicted edges to calculate the bandgap, *E*_g_ = CBM – VBM, similar degeneracy problems emerge; negative bandgap predictions are observed, and metallic MXenes are misclassified as semiconductors. Consequently, the overall accuracy and correlation metrics remained comparable to those of the previous approach, as illustrated in Figure S4 of the SI.

### 3.3 Combining Classification and Regression

To mitigate the misclassification of metallic systems as semiconductors, we devised a two-step approach inspired by previous approaches on inorganic solids.^51^ First, a classification model is trained to differentiate between metallic and semiconducting MXenes. Then, for MXenes identified as semiconductors, a separate regression model —trained exclusively on semiconducting MXenes— is employed to estimate their bandgap values. This method aims to enhance the accuracy of predicting semiconducting properties while reducing misclassification of metallic systems. Again, different classifier ML algorithms were studied, including gradient boosting classifier (GBC), random forest classifier (RFC), support vector classifier (SVC), multilayer perceptron classifier (MLPC), and logistic regression (LR).

The evaluation of the classification models is summarized in Table S4 of the SI, with the corresponding confusion matrices and ROC curves presented in Figures S5 and S6 of the SI, respectively. For more information about the evaluation metrics, we refer to Section S1 and Figure S1 of the SI. DOS-trained models continue to exhibit slightly better performance compared to those using only elemental features. However, at variance from the regression tasks, the difference is marginal, and in some cases such as SVC, the performance is slightly worse. It is worth noting that the models trained solely on elemental features, while generally less accurate, offer the computational advantage of not requiring DOS-based descriptors, which rely on further calculations, making these features more accessible for large-scale screening.

Tree-based models, once again, demonstrate superior performance also in classification tasks, with accuracy of 91% and 92%, precision of 91% and 86%, and area under the curve (AUC) values of 0.97 and 0.95 for RFC and GBC, respectively. All models exhibit strong precision in identifying metallic MXene compounds. However, they encounter more challenges in accurately predicting semiconducting cases, with some semiconductors being misclassified as metallic. Among the evaluated models, GBC stands out for its higher recall. This metric, which quantifies the proportion of actual semiconductors correctly identified by the model, is especially important in this context. A higher recall minimizes the risk of prematurely discarding potential semiconducting MXenes that may be valuable later for photocatalytic applications. By ensuring that more semiconductors are retained in the screening process, the recall allows for a broader exploration of candidate materials. Once these materials are identified, further calculations can refine the selection. This characteristic makes GBC a better suited classification model for the present goals.

For the subsequent regression step, the best-performing model was the RFR, trained with DOS features, which achieved an MAE of 0.24 eV and *R* of 0.92. The results for all tested models are summarized in Table S5 and Figure S7 of the SI. Based on the previous analysis, the final pipeline integrates the GBC classifier and the RFR regressor. Using this setup, a MXene is first classified as metallic or semiconducting *via* GBC, and for those predicted as semiconductors, the bandgap is then estimated using the RFR. As shown in Figure 3, this two-step approach significantly reduces the misclassification of metallic and low-bandgap MXenes, resulting in more accurate and less dispersed predictions, achieving an overall MAE of 0.17 eV and *R* of 0.95, being quite accurate and reliable for a rapid screening. The learning curves in Figure S8 of the SI further illustrate the performance of the regression model. As expected, the error decreases with increasing training data, indicating an improved model performance. The small but consistent gap between the test and training errors suggests that the model is generalized well without significant overfitting. Additionally, the steady decline of the test error implies that further improvements could be achieved with larger data sets.

One concern with this model is that it still requires some DFT calculations to obtain the PBE-level features. However, we note that the required PBE-level calculation and ML model prediction are relatively inexpensive compared with PBE0 calculations. Based on our benchmarks from the La-MXenes screening discussed later, the combined PBE structure optimization and DOS calculation takes ∼8 min on average per structure using 24 CPUs, while the ML model prediction itself (using the GBC + RFR approach) takes less than 1 s once the PBE data is available. In contrast, a single PBE0 DOS calculation can take ∼5 h on average on the same hardware (24 CPUs), and up to 12 h for more complex structures such as *n* = 3 MXenes. This translates to a ∼ ×38 speedup when using our ML approach compared to PBE0 calculations.

With this in mind, the trade-off between model performance and feature accessibility was further evaluated, investigating a simplified version of the classifier-regressor pipeline using only easily obtainable elemental features, those requiring *per se* neither structural modeling nor DFT calculations. These included periodic table-derived properties such as the atomic number *Z*, group, period, electronegativity, electronic affinity, van der Waals and atomic radii, plus MXene *n* index, stacking, and hollow site. The classifier achieved an accuracy of 86%, slightly smaller than that of the full-feature model of 92%. However, its precision dropped to 70%, with a higher rate of false positives. When combined with the regressor, the overall model yielded a worse *R* coefficient of 0.80 and a less accurate MAE of 0.30 eV compared to the full model of 0.17 eV. While these results are notably less accurate and more dispersed than those obtained with the full feature set, they still demonstrate a decent predictive power given the minimal input requirements.

A comparison is made as well with respect the previous work of Rajan *et al.* (ref 32), who also developed ML models for the MXene bandgap prediction. Such earlier ML models were appealing since some did not rely on either PBE bandgaps or DOS inputs, which reduced the computational need. As far as the predictive performance is concerned, the classifier reached a similar accuracy of 94% compared to the present 92%, and the regressor had *R*/MAE of 0.91/0.11 eV vs the present 0.95/0.17 eV. Still, a key distinction lies in the generality and scope of the training data; Rajan *et al.* had the classifier and regressor trained exclusively on *n* = 1 MXenes, with the regressor trained with a limited data set of 70 systems, composed exclusively by Sc- and Y-based MXenes, which explains the good performance, knowing that composition biases the results. In contrast, our model was developed on a significantly broader and more chemically diverse data set over 4000 MXenes, which offers a solid basis for the generalizability and applicability of the present ML model while still achieving comparable accuracy metrics.

The nature of the random forest algorithm, for either the classification or the regression, can provide the importance of the features used in the model. By aggregating the information gained across all trees in the forest, the algorithm identifies which features are most effective at separating the data or predicting the bandgap. Here, we extracted the feature importance with the RFC and RFR models, trained with elemental features alone and also including DOS features, as seen in Figure 4. Moreover, we considered a version trained with only periodic table features. To classify the MXene into metallic or semiconductor, the DOS bins near the Fermi energy (where DOS_51_ is the bin at the Fermi level) exhibit the highest importance, emphasizing their influence on electronic and conducting properties, while the PBE bandgap also significantly impacts predictions. When relying solely on elemental features for classification and regression tasks, structural factors, such as the MXene width, *d*, and interatomic distances were more influential than elemental descriptors, such as the termination electronic affinity, EA­(T), and electronegativity, EN­(T), or the metal atomic number, *Z*, and group.

**Figure 4:**
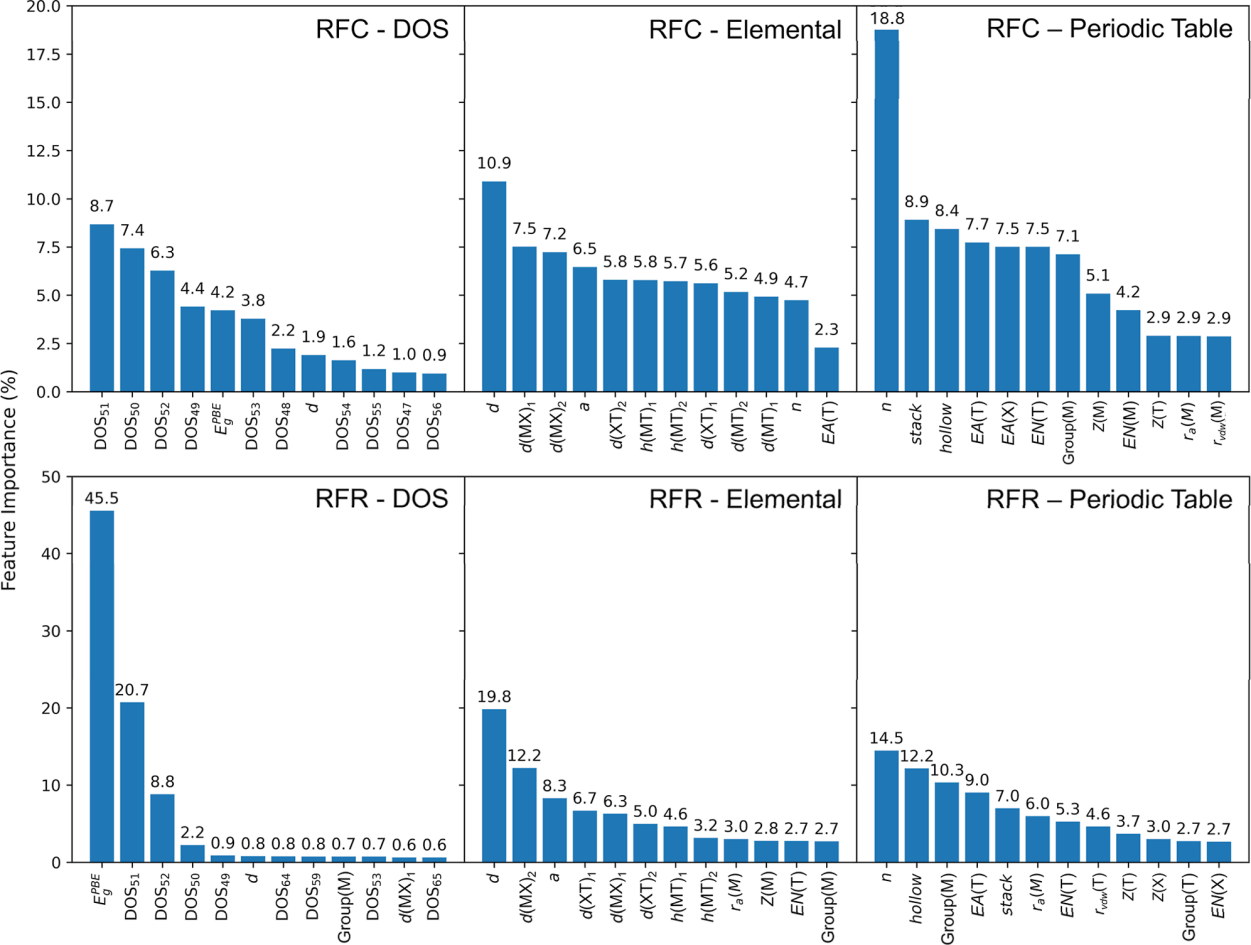
Feature importance derived with the RF algorithm. The top panel represent the importance values extracted from the RFC model, while the bottom one using RFR. From left to right, the plots display the importance values from models trained with DOS features included, with only elemental features, and with only periodic table features.

When considering only periodic table features, the MXene *n* index shows the highest importance in both the classification and regression models, followed by the stacking and hollow phases of the MXene. In the regression, when DOS features were included, the PBE bandgap stood out as the most significant feature, followed by DOS bins around the Fermi level. This observation is consistent with expectations, as PBE gaps are known to correlate strongly with PBE0 gaps, as demonstrated, for instance, in metal–organic frameworks, exhibiting an *R* coefficient of 0.96,^52^ and also confirmed in MXenes with our own data (see Figure S9 of the SI). This analysis underscores the necessity of integrating computed data, including PBE-derived values and DOS features, alongside elemental properties, in order to enhance ML model performance. While this strong correlation may suggest that a simple linear scaling could approximate a PBE0 gap from a PBE value, such approaches are limited to semiconducting systems with nonzero PBE bandgaps (*cf.* Figure S9 of the SI). When metallic cases are included, the correlation breaks down, and scaling becomes unreliable. Furthermore, the relationship is sensitive to structural and chemical variations, such as surface terminations and layer numbers, making its general use difficult. The present ML models tackle these limitations by accurately classifying metallic *vs.* semiconducting cases and refining predictions across a chemically diverse data set —capabilities that cannot be reliably achieved using a single scaling rule.

### 3.4 MXgap: A Python Package for MXene Bandgap Prediction

Based on our trained models, an open-source Python package, MXgap, was designed to streamline MXene bandgap predictions. Featuring a user-friendly command-line interface, the program processes output files from a VASP calculation (primarily the final, optimized structure, plus the DOS file), automatically extracting and parsing all the needed features, employing the pretrained models to predict the bandgap of a given MXene structure. By default, the tool utilizes the best-performing model mentioned —a combination of a classifier and a regressor— but any of the discussed models can be selected. The package is freely available on GitHub and the Python Package Index (PyPI), where more detailed documentation and examples are provided.

### 3.5 Screening La-Based MXenes

To demonstrate the practical application of our models, we applied the optimized GBC + RFR model to screen 396 novel La-based MXenes for their bandgap properties, as depicted in Figure 5a, aiming to identify new promising photocatalysts. La-MXenes were selected because, among the already synthesized MXenes, group III-based MXenes (containing Sc and Y) were already known,^53,54^ and theoretical studies posed them as promising candidates for water splitting photocatalysis,^55^ making La a promising candidate for further exploration. To evaluate the position of these new La-based MXenes within the existing chemical space, we employ the *t*-distributed stochastic neighbor embedding (*t*-SNE) method. This dimensionality reduction technique reveals that La-based MXenes form a distinct cluster, separate from both the rest of the data set and other group III MXenes; see Figure S10 of the SI. This indicates that the model perceives these materials as genuinely novel, making predictions more challenging due to the need for extrapolation beyond the trained patterns. From the screened structures, the model predicted 270 semiconductor cases, 73 of which had *E*_g_ > 1.23 eV, which is a requisite for the photocatalyzed water splitting process. Since we are considering six terminated structures for each MXene, we selected the most stable one, reducing the number of optimal cases from 73 to 14. To validate the model, we selected 14 more random cases and performed PBE0 calculations to get their bandgap and compared it to the ML prediction.

**Figure 5:**
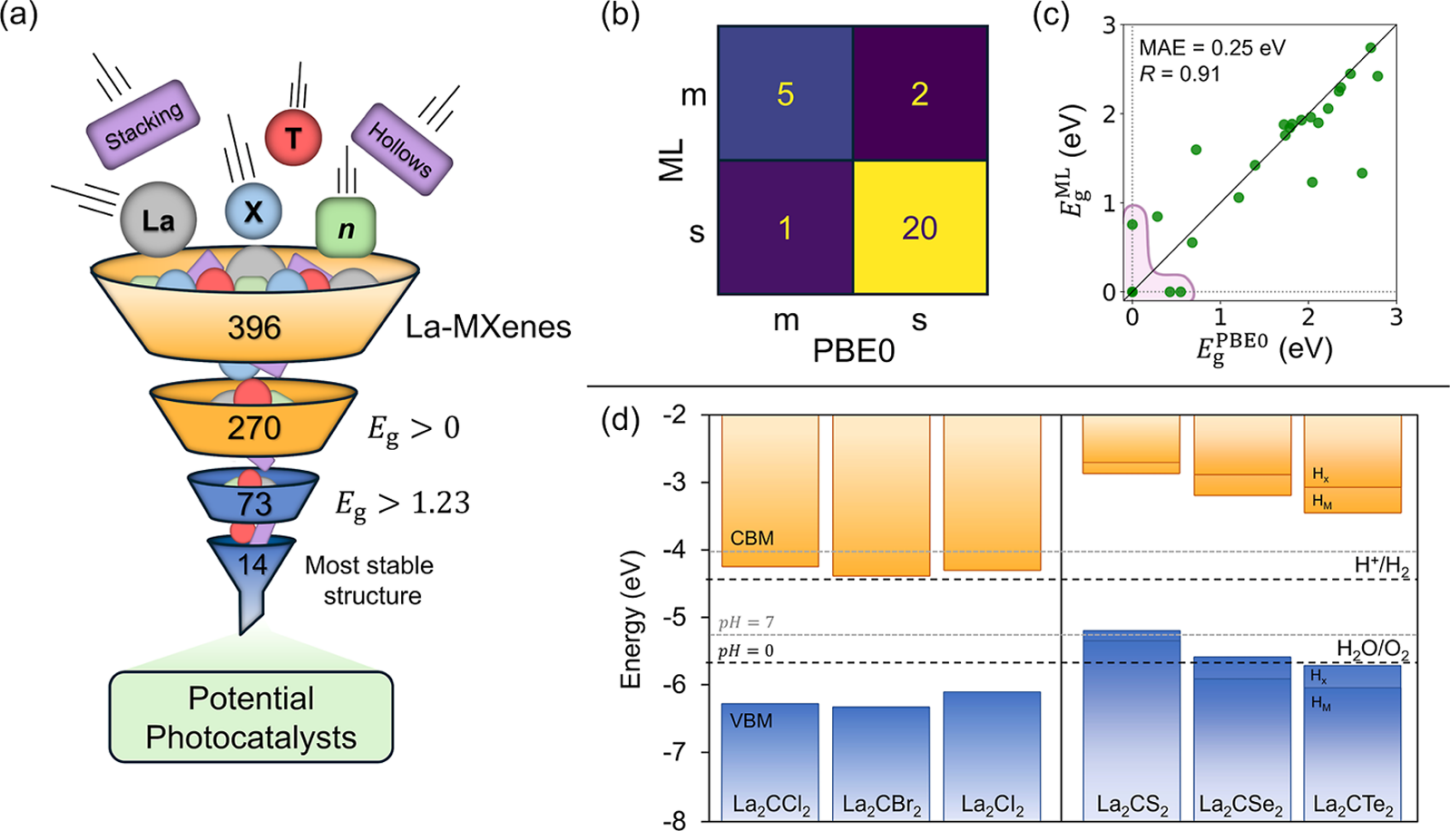
Screening of La-based MXenes for their photoactive properties. (a) Schematic representation of the screening using the best obtained ML model. Out of an initial set of 396 La-MXenes, 14 emerged as promising photocatalysts. (b) Confusion matrix between the true (PBE0) and predicted (ML) metallic (m) or semiconductor (s) predictions of the classifier model for the tested La-based MXenes. (c) Correlation plot between the PBE0 bandgap, *E*_g_^PBE0^, and the predicted bandgap, *E*_g_^ML^, both in eV, for the tested La-based cases. The purple area marks the cases the classifier misclassifies, and the ones it predicts its metallic behavior correctly (the eight cases in purple of the confusion matrix). (d) Band alignment diagrams relative to the H^+^/H_2_ and H_2_O/O_2_ redox potential energy levels (dotted lines), for the six cases with correct band alignment identified through the screening. The blue and orange bars indicate the VB and CB, respectively. For the Janus chalcogen-terminated MXenes, the band edge position is represented for each surface (H_M_ or H_X_).

The classifier (see Figure 5b) presents an accuracy of 89%, which demonstrates the strength of the model at discriminating metallic from semiconductor MXenes, even those of an external data set. For the regression, the results, shown in Figure 5c and Table S6 of the SI, present good agreement between ML and PBE0 bandgaps, with a MAE of 0.25 eV and *R* = 0.91. These strong correlations are particularly noteworthy given that they involve extrapolation, as highlighted by the earlier *t*-SNE analysis. For lower bandgap values, there is a higher variability in predictions compared to those with larger ones, for which predictions tend to be more accurate. Two clear cases stand out, La_2_CO_2_ and La_2_CTe_2_, where the predicted bandgap is notably lower than the PBE0 value. This discrepancy can be attributed to their smaller PBE bandgap, which, as previously observed, is identified by the model as a significant feature. Consequently, this feature biases the predicted bandgap, resulting in a lower-than-expected value. Moreover, Te-terminated MXenes are predominantly metallic or present a small bandgap, which can bias the predicted bandgap results. We tested the program with alternative models (GBC + KRR and GBC + GBR) to assess their performance, as presented in Table S6 of the SI. While these models exhibited slightly lower accuracy, they still captured the main trends, with most predicted values remaining comparable, though with a higher MAE.

For the 14 optimal cases from the screening mentioned before, PBE0 electronic structure calculations were carried out to also gain their band alignment with respect to the water splitting reaction. Finally, six MXenes —La_2_CT_2_ (T = Cl, Br, I, S, Se, Te)— presented a suitable band alignment, as shown in Figure 5d (the remaining nonsuitable ones can be found in Figure S11 of the SI). The resulting promising La-MXenes, correlate well with other adequate group III based MXenes, which present the same halide or chalcogen terminations.^55^ The S-, Se-, and Te-terminated MXenes adopt an ABC H_MX_ structure, which renders them as Janus materials with distinct band alignments on each face. For the La_2_CT_2_ (T = Cl, Br, I, Se, Te) cases, the overall water splitting photocatalysis seems to be possible at *pH* = 0, while La_2_CS_2_ and the H_X_ face of La_2_CSe_2_ are limited to photocatalyzing the HER process. When increasing the *pH* to 7, the CBM of the halide-terminated MXenes falls below the H^+^/H_2_ reduction potential, making them suitable only for photocatalyzing the OER. In contrast, for the chalcogen-terminated systems, the increase in *pH* is beneficial since it enables La_2_CSe_2_ and La_2_CS_2_ H_X_ face to potentially perform the overall water splitting. It is important to note that while our analysis incorporates the *pH*-dependent shift of the water redox potentials, it does not consider possible VBM and CBM position changes with *pH* and local interfacial conditions.^56,57^ Therefore, while the computed band alignments provide a useful initial screening criterion, experimental validation and more detailed interfacial modeling would be advised to fully confirm the photocatalytic viability of the proposed candidates.

An effective photocatalyst must be capable of absorbing a substantial portion of either visible or UV light —the primary components of solar radiation— and efficiently converting this absorbed light into hole–electron photogenerated pairs, which eventually separate and promote the photocatalytic process. Here, we explored the light harvesting properties of this new six promising MXenes by determining the optical absorption coefficient (eq 1); see Figure 6. Most MXenes demonstrate strong absorption in the visible spectrum, with their major absorption peaks occurring within this region. An exception is La_2_CS_2_ where the first two prominent absorption peaks are located in the IR and UV regions. Halogen-terminated MXenes exhibit their initial absorption peak in the IR region but maintain significant absorption within the visible range. Notably, La_2_CSe_2_ and La_2_CTe_2_ stand out by presenting their first major absorption peaks directly in the visible spectrum.^55^

**Figure 6:**
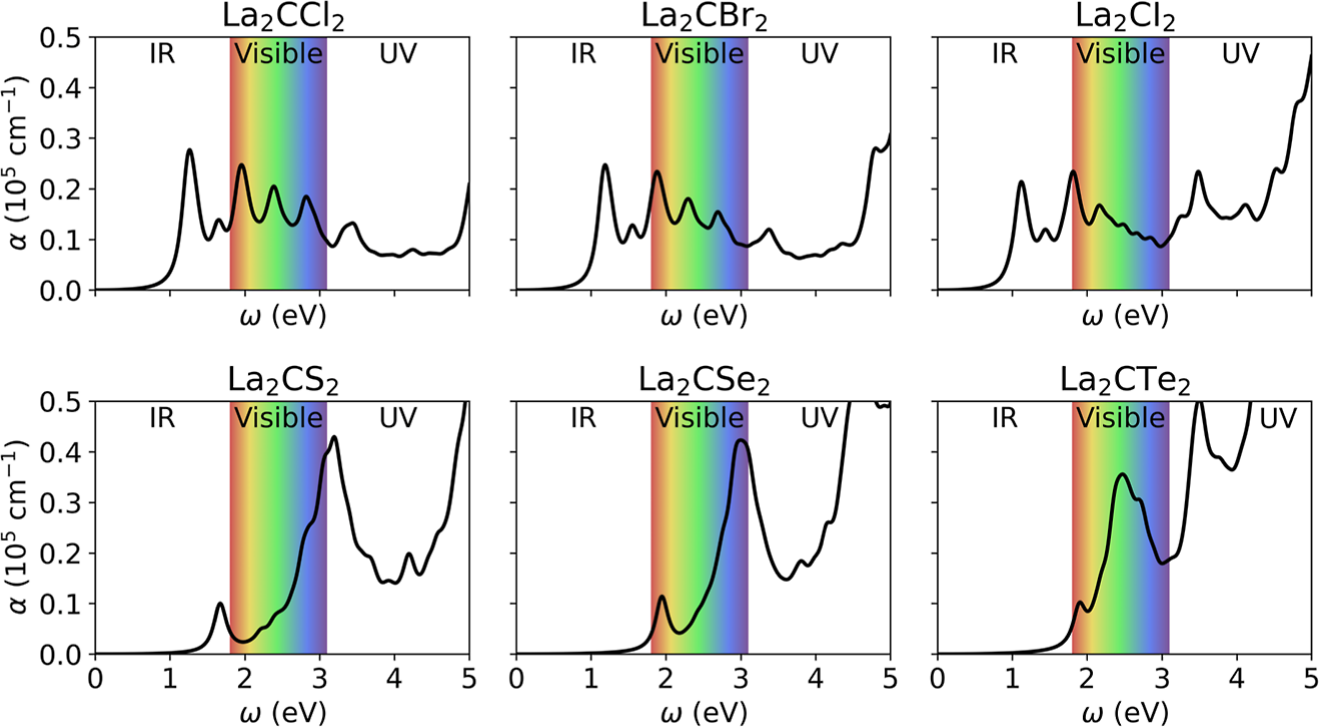
Optical absorption coefficient, α, in 10^5^ cm^–1^ units, as a function of the photon energy, ω, in eV, for the six found promising La-based MXenes.

The upper limit of the STH efficiency has also been estimated at *pH* = 0, with the results and the parameters needed for its calculation listed in Table 1. It is observed how the light absorption efficiency, η_abs_, has a close relationship with its bandgap, since a bandgap increase is accompanied by a decrease in light absorption efficiency. The halide-terminated MXenes exhibit the highest absorption efficiency and carrier utilization efficiency, resulting in superior STH efficiencies, ranging from 43–47%. On the contrary, chalcogen-terminated MXenes, due to their larger optical gap, show lower absorption and STH efficiencies, in the 6–14% range. This discrepancy between halogen and chalcogen terminations is also observed for Sc- and Y-based MXenes.^55^ In summary, six new potential photocatalysts have been discovered and further studied. The application of this ML approach has significantly reduced the number of hybrid functionals and advanced DFT calculations, thereby accelerating the process of identifying promising MXenes for water splitting photocatalysis.

**Table 1:** Parameters for the STH Efficiency Evaluation

	Ε_opt_	Δϕ	χ_H_2__	χ_O_2__	η_cu_	η_abs_	η_STH_	η_STH_^′^
L_a2_CCl_2_	1.22	0	0.18	0.62	75.2	62.1	46.7	46.7
La_2_CBr_2_	1.15	0	0.03	0.66	79.3	54.9	43.5	43.5
La_2_CI_2_	1.09	0	0.11	0.44	81.4	53.4	43.4	43.4
La_2_CS_2_ ^a^	1.66	0.15	1.24	0.08	53.1	26.3	13.4	13.0
La_2_CSe_2_	1.94	0.30	1.40	0.23	39.3	27.2	10.7	10.2
La_2_CTe_2_	1.90	0.36	1.17	0.38	41.2	36.4	15.0	14.1

a Results at *pH* = 7.

*E*_opt_ is the optical gap, Δϕ is the difference between the vacuum energies at the two MXene surfaces for Janus cases, χ_H_2__ and χ_O_2__ are the HER and OER overpotentials at *pH* = 0, respectively, all given in eV, and η_abs_, η_cu_, η_STH_, and η_STH_^′^ represent the efficiency of light absorption, carrier utilization, STH, and corrected STH, respectively, given as a percentage.

## 4 Conclusions

This study contributes to the broader understanding of MXenes as promising materials for various clean energy applications by utilizing ML to predict their bandgap properties. The bandgap is a key factor influencing the material performance across a range of applications, including photocatalysis, where we focused on. Using a comprehensive data set of 4356 MXene structures, generated from a computational screening grounded in hybrid DFT calculations, we developed and validated multiple ML models. The best approach consists of a classifier-regressor pipeline, achieving a high classification accuracy of 92% for distinguishing metallic from semiconducting MXenes and a low MAE of 0.17 eV for bandgap prediction. Additionally, the inclusion of physically interpretable descriptors in the ML models, combined with feature importance analysis, provided valuable insights into the key properties influencing the semiconducting behavior and bandgap values of MXenes. The study highlights how incorporating PBE-derived DOS information significantly enhances prediction accuracy and underscores the greater importance of structural parameters over elemental features derived from the periodic table.

The trained models have been integrated into an open-source Python package called MXgap, which, to the best of our knowledge, is the first tool accessible to all users that utilizes pretrained ML models for MXene bandgap predictions. While a few other studies have developed ML models for MXene bandgap predictions, they do not offer such user-friendly tools, which hinders new users from efficiently making quick and accurate predictions. To validate the developed best model, we applied it to screen 396 novel La-based MXene structures, which led to six optimal candidates —La_2_CCl_2_, La_2_CBr_2_, La_2_CI_2_, La_2_CS_2_, La_2_CSe_2_, and La_2_CTe_2_— which were evaluated through advanced DFT methods. The results highlighted their suitable band alignments and strong light absorption properties in the visible region, with STH efficiencies reaching 47% for halide-terminated structures.

This study not only paves the way for the identification of promising MXenes for photocatalytic applications but also establishes a methodology that can be extended to other MXene bandgap-dependent properties and applications, including photovoltaics and energy storage. Future efforts could focus on expanding the data set to include more diverse compositions and refining the ML models to increase their accuracy.

## Supplementary Material



## Data Availability

The database used to train the models can be found at 10.5281/zenodo.14858915. Any additional data will be shared upon request. Code availability statement: The MXgap software to estimate the bandgap of MXenes is available at https://github.com/diegonti/mxgap.

## Notes

Features information, definition, information and evaluation metrics for all the trained models and approaches, *t*-SNE projection of the chemical space, validation results for the La-based MXenes, and band alignments for the nonsuitable cases (PDF)

## References

[1] Larcher D., Tarascon J.-M. (2015). Towards Greener and More Sustainable Batteries for
Electrical Energy Storage. Nat. Chem..

[2] Zhu B., Fan L., Mushtaq N., Raza R., Sajid M., Wu Y., Lin W., Kim J.-S., Lund P. D., Yun S. (2021). Semiconductor Electrochemistry
for Clean Energy Conversion and Storage. Electrochem.
Energy Rev..

[3] Richter R., Caillol S. (2011). Fighting Global Warming:
The Potential of Photocatalysis
against CO_2_, CH_4_, N_2_O, CFCs, Tropospheric
O_3_, BC and Other Major Contributors to Climate Change. J. Photochem. Photobiol..

[4] Wang G., Chang J., Tang W., Xie W., Ang Y. (2022). 2D Materials
and Heterostructures for Photocatalytic Water-Splitting: A Theoretical
Perspective. J. Phys. D: Appl. Phys..

[5] Shang Z., Feng X., Chen G., Qin R., Han Y. (2023). Recent Advances
on Single-Atom Catalysts for Photocatalytic CO_2_ Reduction. Small.

[6] Morales-García Á., Viñes F., Sousa C., Illas F. (2023). Toward a Rigorous Theoretical
Description of Photocatalysis Using Realistic Models. J. Phys. Chem. Lett..

[7] Yi Y., Chen X., Zhao Y., Xu X., Zhang P., Li C. (2025). MXene-Based
Semiconductor Materials for Various Applications in Photocatalysis
Field. Energy Technol..

[8] Naguib M., Kurtoglu M., Presser V., Lu J., Niu J., Heon M., Hultman L., Gogotsi Y., Barsoum M. W. (2011). Two-Dimensional
Nanocrystals Produced by Exfoliation of Ti_3_AlC_2_. Adv. Mater..

[9] Gogotsi Y., Anasori B. (2019). The Rise of MXenes. ACS Nano.

[10] Downes M., Shuck C. E., Lord R. W., Anayee M., Shekhirev M., Wang R. J., Hryhorchuk T., Dahlqvist M., Rosen J., Gogotsi Y. (2023). M_5_X_4_: A Family
of MXenes. ACS Nano.

[11] Alhabeb M., Maleski K., Anasori B., Lelyukh P., Clark L., Sin S., Gogotsi Y. (2017). Guidelines for Synthesis and Processing of Two-Dimensional
Titanium Carbide (Ti_3_C_2_T_x_ MXene). Chem. Mater..

[12] Naguib M., Mashtalir O., Carle J., Presser V., Lu J., Hultman L., Gogotsi Y., Barsoum M. W. (2012). Two-Dimensional
Transition Metal Carbides. ACS Nano.

[13] Hope M. A., Forse A. C., Griffith K. J., Lukatskaya M. R., Ghidiu M., Gogotsi Y., Grey C. P. (2016). NMR Reveals the
Surface Functionalization of Ti_3_C_2_ MXene. Phys. Chem. Chem. Phys..

[14] Kamysbayev V., Filatov A. S., Hu H., Rui X., Lagunas F., Wang D., Klie R. F., Talapin D. V. (2020). Covalent Surface
Modifications and Superconductivity of Two-Dimensional Metal Carbide
MXenes. Science.

[15] Ding H., Li Y., Li M., Chen K., Liang K., Chen G., Lu J., Palisaitis J., Persson P. O. Å., Eklund P., Hultman L., Du S., Chai Z., Gogotsi Y., Huang Q. (2023). Chemical Scissor-Mediated
Structural Editing of Layered Transition Metal Carbides. Science.

[16] VahidMohammadi A., Rosen J., Gogotsi Y. (2021). The World
of Two-Dimensional Carbides
and Nitrides (MXenes). Science.

[17] Gogotsi Y., Anasori B. (2023). The Global Expansion of MXenes. Graphene 2D Nanomater..

[18] Pang J., Mendes R. G., Bachmatiuk A., Zhao L., Ta H. Q., Gemming T., Liu H., Liu Z., Rümmeli M. H. (2019). Applications
of 2D MXenes in Energy Conversion and Storage Systems. Chem. Soc. Rev..

[19] Huang L., Ding L., Caro J., Wang H. (2023). MXene-Based Membranes
for Drinking Water Production. Angew. Chem.,
Int. Ed..

[20] Dolz D., De Armas R., Lozano-Reis P., Morales-García Á., Viñes F., Sayós R., Illas F. (2024). Understanding the Reverse
Water-Gas Shift Reaction over Mo_2_C MXene Catalyst: A Holistic
Computational Analysis. ChemCatChem.

[21] Ontiveros D., Viñes F., Sousa C. (2023). Bandgap Engineering
of MXene Compounds
for Water Splitting. J. Mater. Chem. A.

[22] Ontiveros D., Vela S., Viñes F., Sousa C. (2024). Tuning MXenes towards
Their Use in Photocatalytic Water Splitting. Energy, Environ. Mater..

[23] Chen W., Pasquarello A. (2012). Band-Edge
Levels in Semiconductors and Insulators:
Hybrid Density Functional Theory *versus* Many-body
Perturbation Theory. Phys. Rev. B.

[24] Jai A. (2024). Machine Learning
in Materials Research: Developments over the Last Decade and Challenges
for the Future. Curr. Opin. Solid State Mater.
Sci..

[25] Schmidt J., Marques M. R. G., Botti S., Marques M. A. L. (2019). Recent Advances
and Applications of Machine Learning in Solid-State Materials Science. npj Comput. Mater..

[26] Mobarak M. H., Mimona M. A., Islam M. A., Hossain N., Zohura F. T., Imtiaz I., Rimon M. I. H. (2023). Scope
of Machine Learning in Materials
Research—A Review. Appl. Surf. Sci. Adv..

[27] Abraham B. M., Jyothirmai M. V., Sinha P., Viñes F., Singh J. K., Illas F. (2024). Catalysis in the Digital Age: Unlocking
the Power of Data with Machine Learning. Wiley
Interdiscip. Rev.: Comput. Mol. Sci..

[28] Gouveia J. D., Galvão T. L. P., Iben Nassar K., Gomes J. R. B. (2025). First-principles
and Machine Learning Approaches for Interpreting and Predicting the
Properties of MXenes. npj 2D Mater. Appl..

[29] Frey N. C., Wang J., Vega Bellido G. I., Anasori B., Gogotsi Y., Shenoy V. B. (2019). Prediction of Synthesis
of 2D Metal Carbides and Nitrides
(MXenes) and Their Precursors with Positive and Unlabeled Machine
Learning. ACS Nano.

[30] Roy P., Rekhi L., Koh S. W., Li H., Choksi T. S. (2023). Predicting
the Work Function of 2D MXenes Using Machine Learning Methods. J. Phys.: Energy.

[31] Abraham B. M., Sinha P., Halder P., Singh J. K. (2023). Fusing a Machine
Learning Strategy with Density Functional Theory to Hasten the Discovery
of 2D MXene-based Catalysts for Hydrogen Generation. J. Mater. Chem. A.

[32] Rajan A. C., Mishra A., Satsangi S., Vaish R., Mizuseki H., Lee K.-R., Singh A. K. (2018). Machine-Learning-Assisted
Accurate
Band Gap Predictions of Functionalized MXene. Chem. Mater..

[33] Mishra A., Satsangi S., Rajan A. C., Mizuseki H., Lee K.-R., Singh A. K. (2019). Accelerated Data-driven
Accurate Positioning of the
Band Edges of MXenes. J. Phys. Chem. Lett..

[34] Zhang Y., Xiong R., Sa B., Zhou J., Sun Z. (2021). MXenes: Promising
Donor and Acceptor Materials for High-Efficiency Heterostructure Solar
Cells. Sustainable Energy Fuels.

[35] Gogotsi Y. (2023). The Future
of MXenes. Chem. Mater..

[36] Pedregosa F., Varoquaux G., Gramfort A., Michel V., Thirion B., Grisel O., Blondel M., Prettenhofer P., Weiss R., Dubourg V., Vanderplas J., Passos A., Cournapeau D., Brucher M., Matthieu P., Duchesnay É. (2011). Scikit-learn: Machine Learning in Python. J. Mach. Learn. Res..

[37] Hohenberg P., Kohn W. (1964). Inhomogeneous Electron Gas. Phys. Rev..

[38] Kohn W., Sham L. J. (1965). Self-consistent Equations Including
Exchange and Correlation
Effects. Phys. Rev..

[39] Kresse G., Hafner J. (1993). *Ab initio* Molecular
Dynamics for Liquid
Metals. Phys. Rev. B.

[40] Blöchl P. E. (1994). Projector
Augmented-Wave Method. Phys. Rev. B.

[41] Perdew J. P., Burke K., Ernzerhof M. (1996). Generalized
Gradient Approximation
Made Simple. Phys. Rev. Lett..

[42] Perdew J. P., Yue W. (1986). Accurate and Simple
Density Functional for the Electronic Exchange
Energy: Generalized Gradient Approximation. Phys. Rev. B.

[43] Adamo C., Barone V. (1999). Toward Reliable Density
Functional Methods without
Adjustable Parameters: The PBE0 model. J. Chem.
Phys..

[44] Monkhorst H. J., Pack J. D. (1976). Special Points for
Brillouin-zone Integrations. Phys. Rev. B.

[45] Hybertsen M. S., Louie S. G. (1986). Electron Correlation in Semiconductors
and Insulators:
Band Gaps and Quasiparticle Energies. Phys.
Rev. B.

[46] Albrecht S., Reining L., Del Sole R., Onida G. (1998). *Ab Initio* Calculation of Excitonic Effects in the
Optical Spectra of Semiconductors. Phys. Rev.
Lett..

[47] Fu C. F., Sun J., Luo Q., Li X., Hu W., Yang J. (2018). Intrinsic
Electric Fields in Two-dimensional Materials Boost the Solar-to-Hydrogen
Efficiency for Photocatalytic Water Splitting. Nano Lett..

[48] Gueymard C. A., Myers D., Emery K. (2002). Proposed Reference
Irradiance Spectra
for Solar Energy Systems Testing. Sol. Energy.

[49] Zheng Y., Jiao Y., Jaroniec M., Qiao S. Z. (2015). Advancing the Electrochemistry
of the Hydrogen Evolution Reaction through Combining Experiment and
Theory. Angew. Chem., Int. Ed..

[50] McCrory C. C. L., Jung S., Peters J. C., Jaramillo T. F. (2013). Benchmarking
Heterogeneous Electrocatalysts for the Oxygen Evolution Reaction. J. Am. Chem. Soc..

[51] Zhuo Y., Mansouri Tehrani A., Brgoch J. (2018). Predicting the Band Gaps of Inorganic
Solids by Machine Learning. J. Phys. Chem. Lett..

[52] Fumanal M., Capano G., Barthel S., Smit B., Tavernelli I. (2020). Energy-based
Descriptors for Photo-catalytically Active Metal–Organic Framework
Discovery. J. Mater. Chem. A.

[53] Meshkian R., Tao Q., Dahlqvist M., Lu J., Hultman L., Rosen J. (2017). Theoretical
Stability and Materials Synthesis of a Chemically Ordered MAX Phase,
Mo_2_ScAlC_2_, and its Two-dimensional Derivative
Mo_2_ScC_2_ MXene. Acta Mater..

[54] Maeda K., Wakayama H., Washio Y., Ishikawa A., Okazaki M., Nakata H., Matsuishi S. (2020). Visible-light-induced
Photocatalytic
Activity of Stacked MXene Sheets of Y_2_CF_2_. J. Phys. Chem. C.

[55] Ontiveros D., Viñes F., Sousa C. (2025). Exploring the Photoactive Properties
of Promising MXenes for Water Splitting. J.
Mater. Chem. A.

[56] Guo Z., Ambrosio F., Chen W., Gono P., Pasquarello A. (2018). Alignment
of Redox Levels at Semiconductor–Water Interfaces. Chem. Mater..

[57] Ambrosio F., Wiktor J., Pasquarello A. (2018). pH-Dependent
Catalytic Reaction Pathway
for Water Splitting at the BiVO_4_–Water Interface
from the Band Alignment. ACS Energy Lett..

