# Supplementary material for: MXgap: A MXene Learning Tool for Bandgap Prediction
Source: ACS Catal. 2025 Aug 5;15(16):14403–13. doi: 10.1021/acscatal.5c04191 (PMC12363268; doi:10.1021/acscatal.5c04191)
Supplement: Supplementary file 1 [file cs5c04191_si_001.pdf]

# MXgap: A MXene Learning Tool for Bandgap Prediction

Diego Ontiveros,[†] Sergi Vela,[‡*] Francesc Viñes,[†*] and Carmen Sousa[†]

[†] *Departament de Ciència de Materials i Química Física & Institut de Química Teòrica i Computacional (IQTCUB), Universitat de Barcelona, c/ Martí i Franquès 1-11, 08028, Barcelona, Spain.*

[‡] *Institut de Química Avançada de Catalunya (IQAC-CSIC), Barcelona, Spain.*

[*] Corresponding authors: Sergi Vela (sergi.vela@iqac.csic.es); Francesc Viñes (francesc.vines@ub.edu)

**Table S1.** Information of the features used for training the machine learning (ML) model. Structural features were selected for both surfaces of the MXene, as the faces can differ in certain configurations, such as in $H_{MX}$ structures, where one surface has terminations in the $H_M$ hollow site and the other in the $H_X$ position (see Fig. 6c of the manuscript). Elemental features were chosen for each atomic species in the MXene.

| Feature | Description |
|---|---|
| ***n*** | MXene *n* index from $M_{n+1}X_nT_2$ |
| **stacking** | Stacking, encoded as a number. ABC = 0, ABA = 1 |
| **hollow** | Hollow site, encoded as a number. $H_M$/H = 0, $H_{MX}$ = 1, $H_X$ = 2 |
| ***a*** | Optimized lattice parameter |
| ***d*** | Optimized width |
| **Structural Features, for both Surfaces** | |
| ***h*(MT)** | Distance between M-T layers |
| ***d*(MT)** | Distance between M-T atoms |
| ***d*(XT)** | Distance between X-T atoms |
| ***d*(MX)** | Distance between M-X atoms |
| **Elemental Features, For each Atom Species (M, X, T)** | |
| **Z** | Atomic number (number of protons) |
| **group** | Group number |
| **row** | Row number |
| **EN** | Electronegativity |
| **EA** | Electron affinity |
| $\boldsymbol{r_{vdW}}$ | Van der Waals (vdW) radius |
| $\boldsymbol{r_a}$ | Atomic radius |
| **Electronic Structure Properties (Density of States – DOS Features)** | |
| **VBM_PBE** | Valence band maximum (VBM) energy with Perdew-Burke-Ernzerhof (PBE) exchange-correlation functional without vacuum correction (so, essentially, the Fermi energy, $E_F$) |
| **CBM_PBE** | Conduction band minimum (CBM) energy with PBE |
| $\boldsymbol{E_g}$**_PBE** | Bandgap energy with PBE |
| **DOS** | Averaged PBE DOS from ±5 eV around the Fermi level. 100 bins in total, $DOS_{51}$ is just before $E_F$. |

## S1. ML Methods

### S1.1 Training and Testing Strategy

Since different ML models are considered (see Section S2), one must regard the different parameters that each model possesses, such as the number of trees in tree-based models (RF or GB) or the type of kernel in kernel-based models (SVM or KRR). These are the so-called model hyperparameters and need to be tuned to optimize the performance of the models. To do so, here a flat five-fold cross-validation strategy was used during the hyperparameter tuning phase of the model training. This is, for each set of hyperparameters, the training set was divided into five equally sized folds: Four folds were used for training, while the remaining one was used for validation, repeating the process five times with different folds. Their performance was then averaged across all five folds, and the hyperparameter configuration yielding the highest average validation score was selected as optimal. After tuning, the final model was trained using the entire training set with the best hyperparameters, and the trained model was then evaluated on a held-out test set, which was never seen neither during training nor cross-validation.

### S1.2 Evaluation

When building ML models, a critical aspect of ensuring their performance and accuracy lies in correctly evaluating their outputs. The choice of evaluation metrics depends on the model specific task —in this case, regression or classification. These metrics allow us objectively comparing the performance of different models, providing valuable insights during hyperparameter tuning, thus ultimately helping in the selection of the best-performing model.

**Regression**

For regression models, two of the most commonly used evaluation metrics —and those employed in this work— are the MAE and $R$. The MAE (Eq. S1) quantifies the average magnitude of errors between the predicted values, $\hat{y}_i$, and the actual values, $y_i$. It provides an intuitive measure of the average prediction error, with lower values indicating better model accuracy. The $R$ coefficient is extracted from the coefficient of determination (Eq. S2), which measures the strength of the linear relationship between predicted and actual values. This way, $R$ = 1, indicates a perfect linear correlation, while $R$ = 0 indicates no linear correlation. Both metrics evaluate how closely the regression predictions align with the true data points, offering insights into the model's training quality and overall performance.

$$\mathrm{MAE} = \frac{1}{n}\sum_{i}^{n} |y_i - \hat{y}_i| \text{(S1)},$$

$$R^2 = 1 - \frac{\sum_i \left(y_i - \hat{y}_i\right)^2}{\sum_i \left(y_i - \bar{y}\right)^2} \text{(S2)}.$$

**Classification**

In classification tasks, many performance metrics are derived from the confusion matrix, a table that summarizes the comparison between predicted and actual labels. For a binary classification problem, such as classifying MXenes as metallic (labelled as 0) or semiconducting (labelled as 1), the confusion matrix resembles Figure S1a. Here, "T" and "F" represent True and False, while "N" and "P" stand for Negative and Positive. In our case, negative indicates metallic MXenes, while positive are semiconductor MXenes. For example, true positives (TP) are cases correctly predicted as positive (semiconductors) by the model, while false negatives (FN) are cases misclassified as negative (metallic) when they are actually positive (semiconductors).

Accuracy measures the proportion of correctly classified instances among all predictions (Eq. S3). While it provides a general idea of the model correctness, it sometimes can be misleading for imbalanced datasets, such as in the present study, where metallic MXenes are more prominent than semiconductors. Precision (Eq. S4) calculates the proportion of instances predicted as positive that are actually positive, which helps to assess the reliability of positive predictions. On the other hand, recall (also known as true positive rate, TPR; Eq. S5) measures the proportion of actual positive instances that are correctly identified by the model, providing insight into the model ability to detect positive cases. In our study, achieving high recall is particularly advantageous because it ensures that MXenes with a bandgap are correctly identified.

Additionally, the ROC curve is often used to visually evaluate a classifier performance across various thresholds, plotting the trade-off between sensitivity (TPR) and specificity (the ability to avoid false positives). The curve shows the relationship between TPR and the false positive rate, FPR (Eq. S6), as seen in Figure S1b. The AUC provides a single scalar value to summarize the model performance based on the ROC curve. A perfect classifier achieves an AUC = 1, while an AUC = 0.5 reflects random guessing, indicating that the model predictions are no better than pure chance.

$$\text{Accuracy} = \frac{T}{T + F} \text{(S3)},$$

$$\text{Precision} = \frac{TP}{TP + FP} \text{(S4)},$$

$$\text{Recall} = \text{TPR} = \frac{TP}{TP + FN} \text{(S5)},$$

$$\text{FPR} = \frac{FP}{FP + TN} \text{(S6)}.$$

**Figure S1.** Evaluation metrics for classification. (a) Confusion matrix example between the predicted and true labels. Here the labels of 0 or "Negative" are associated to metallic MXenes, while 1 or "Positive" correspond to semiconductor MXenes. (b) ROC curve example. The diagonal black dashed line referees to random classification (AUC = 0.5).

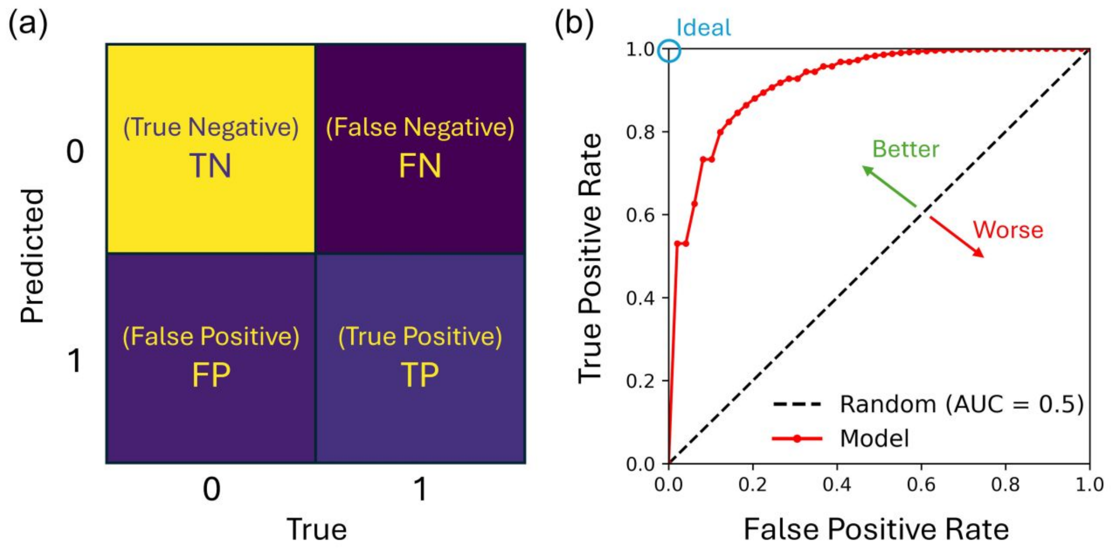

## S2. ML Models

We employed a set of widely used ML models for both classification and regression tasks. All models were implemented using the Scikit-Learn library[1], which provides robust and standardized tools for ML workflows in Python. In most cases, the same algorithmic framework can be used for both classification and regression, such as Random Forest Classifier/Regressor (RFC/RFR). While the core learning approach remains the same across both tasks, these models optimize different loss functions ($\ell$) depending on the objective. Classification models typically minimize logarithmic loss (log-loss) to improve class probability estimates ($\hat{p}_i$), see Eq. S7, whereas regression models aim to minimize squared error (mean squared loss), as in Eq. S8, to predict continuous values. This distinction in loss functions shapes how each model learns from the data and assesses its performance, despite sharing a similar underlying structure and training process. Below we describe each model's learning principle, training objective, and how predictions are aggregated.

$$\ell_C = -\frac{1}{n}\sum_{i=1}^{n}[y_i \ln(\hat{p}_i) + (1 - y_i)\ln(1 - \hat{p}_i)] \text{(S7)},$$

$$\ell_R = \frac{1}{n}\sum_{i=1}^{n}(y_i - \hat{y}_i)^2 \text{(S8)}.$$

**Random Forest (RF)**

It is an ensemble method that constructs a large number of decision trees during training. Each tree is trained on a random subset of the training data selected using bootstrap sampling (random sampling with replacement) and a random subset of features. During training, each tree learns a series of decision rules that split the data based on feature thresholds to best separate the target variable. For classification, predictions are made by taking a majority vote across all trees. For regression, the final prediction is the average of all tree outputs.

**Gradient Boosting (GB)**

This approach is also an ensemble method. Unlike RF, which trains trees independently, GB builds trees one after another. Each new tree is trained to minimize the error (residuals) made by the combined ensemble of previous trees. This is done by fitting the new tree to the negative gradient of the loss function —hence the name "gradient boosting". At prediction time, the outputs of all the trees are summed to produce the final regression value. For classification, initial probabilities are computed in a similar manner, and this probability is then used to determine the final class label.

**Support Vector Machine (SVM)**

SVMs are margin-based models. In classification, the goal is to find the best decision boundary between classes, by finding a hyperplane that maximally separates the classes. The data points that lie closest to the decision boundary —called support vectors— play a key role in defining this boundary. For regression, the

model tries to find a function that predicts target values within a certain margin of tolerance around the true values, while minimizing the complexity of the model. Only data points that lie outside this margin contribute to the model error function and influence the final prediction. SVMs can also use kernel functions to transform data into a higher-dimensional space, allowing them to learn non-linear patterns without explicitly computing the transformation, known as the "kernel trick".

**Multilayer Perceptron (MLP)**

This model consists of a type of artificial neural network composed of an input layer, one or more hidden layers, and an output layer. Each layer consists of nodes (called neurons) that apply a weighted sum of their inputs followed by a non-linear activation function (*e.g.*, *ReLU* = max(0,$x$), sigmoid = $1/(1+e^{-x})$). During training, MLPs use backpropagation combined with gradient descent to adjust the weights of the connections between neurons. This iterative process minimizes the loss function by propagating the prediction error backward through the network. In classification tasks, the final layer typically uses a sigmoid activation to produce probabilities over classes. In regression, the output layer is linear, predicting continuous values.

**Logistic Regression (LR)**

Despite the name, LR is strictly a classification algorithm. It computes a linear combination of the input features —similar to linear regression— but instead of producing a continuous output, the result is passed through a logistic (sigmoid) function. This function maps the value to a range between 0 and 1, representing the predicted class probability. During training, the model uses optimization algorithms like gradient descent to minimize a loss function (usually log-loss), adjusting the weights assigned to each feature. When predicting, LR calculates the probability of each class and selects the one with the highest probability.

**Kernel Ridge Regression (KRR)**

This method combines two concepts: ridge regression and kernel methods. Ridge regression introduces a regularization term to penalize large coefficients, which helps prevent overfitting in models with many correlated features. The kernel component enables the model to learn non-linear relationships by mapping the input datapoints into a high-dimensional space where linear regression can be performed. Training involves solving a regularized least squares problem, which can be computed efficiently thanks to kernel tricks like the radial basis function or polynomial kernels. KRR does not require iterative training like neural networks —it relies on matrix operations for an exact solution. During prediction, KRR computes the similarity between new inputs and training data points via the kernel function, and uses these to make a weighted sum of the target values.

**t-Distributed Stochastic Neighbour Embedding (t-SNE)**

t-SNE is a non-linear dimensionality reduction technique widely used for visualizing high-dimensional data in two or three dimensions. It is especially effective for uncovering patterns, clusters, and relationships in datasets where the underlying structure may not be immediately obvious. Unlike supervised learning

models, such as those mentioned above for classification or regression, t-SNE is unsupervised and does not require target values. Instead of making predictions, it focuses on preserving the local structure of the data: points that are close together in the high-dimensional space are mapped to nearby points in the lower-dimensional projection, while distant points remain relatively far apart.

**Figure S2.** Correlation plots between the true PBE0, $E_{\mathrm{g}}^{\mathrm{PBE0}}$, and the ML predicted bandgaps, $E_{\mathrm{g}}^{\mathrm{ML}}$, using the full database, for each studied model. The top row is using only elemental features, while the bottom row is also including DOS features. Blue and green represent the training and test datasets, respectively. Insets show the mean absolute error (MAE) values, given in eV, and the correlation coefficient, *R*. The studied ML models are gradient boosting regressor (GBR), random forest regressor (RFR), support vector regressor (SVR), multi-layer perceptron regressor (MLPR), and kernel ridge regressor (KRR).

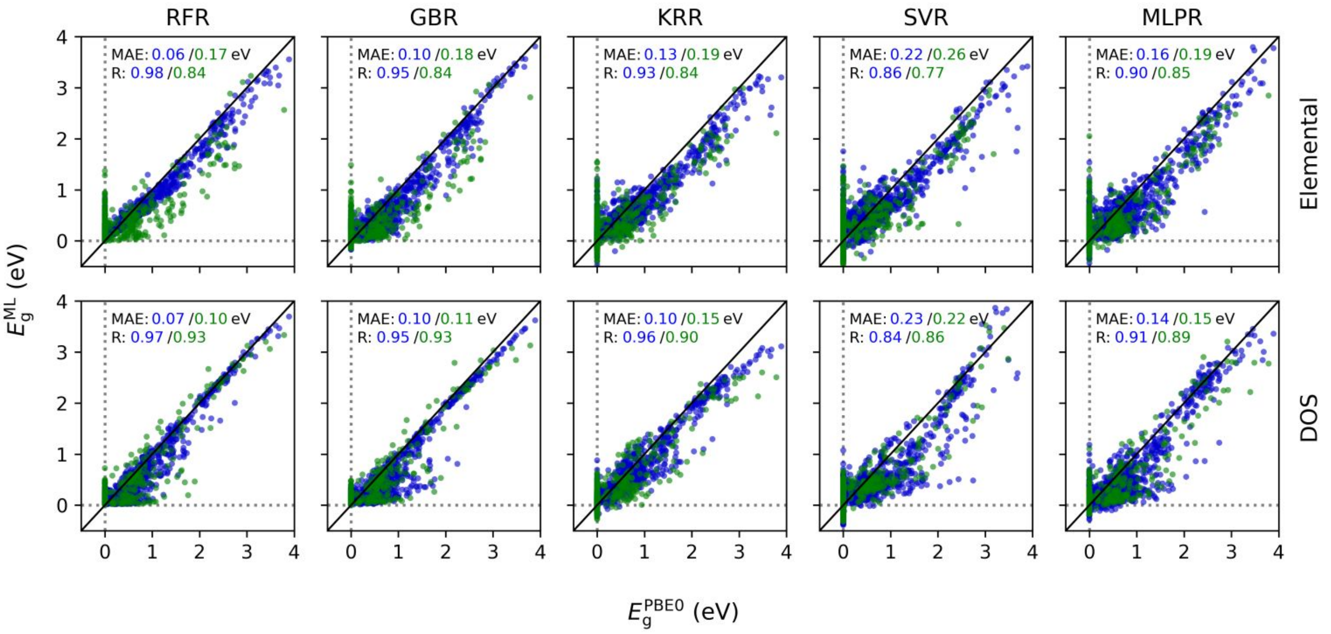

**Table S2**. Evaluation metrics for the test set, obtained with different models using the full database and a 60/20/20 train/validation/test split. The *R*, MAE (in eV), and number of cases with an absolute error larger than 1 eV, $N_{\varepsilon>1}$ (out of 872 test structures), are displayed for two independently-trained models, one with only elemental features and another that also includes DOS features. The $N_{\varepsilon>1}$ also includes the percentage of cases it corresponds, between parentheses.

| | **Elemental** | | | **DOS** | | |
|---|---|---|---|---|---|---|
| **Model** | ***R*** | **MAE / eV** | $N_{\varepsilon>1}$ | ***R*** | **MAE / eV** | $N_{\varepsilon>1}$ |
| **RFR** | 0.84 | 0.17 | 14 (1.6%) | 0.93 | 0.10 | 5 (0.6%) |
| **GBR** | 0.84 | 0.18 | 12 (1.4%) | 0.93 | 0.11 | 4 (0.5%) |
| **KRR** | 0.84 | 0.19 | 12 (1.4%) | 0.90 | 0.15 | 7 (0.8%) |
| **SVR** | 0.77 | 0.26 | 15 (1.7%) | 0.86 | 0.22 | 8 (0.9%) |
| **MLPR** | 0.85 | 0.19 | 12 (1.4%) | 0.89 | 0.15 | 10 (1.1%) |

**Table S3.** Evaluation metrics for the different models for predicting the valence band maximum (VBM), conduction band minimum (CBM), and bandgap, $E_g$, using the full database. The $R$, MAE (in eV), and $N_{\varepsilon>1}$ are displayed for two independently-trained models, one with only elemental features and another that also includes DOS features. The $N_{\varepsilon>1}$ also includes the percent of cases it corresponds, between parentheses.

| | | VBM | | | CBM | | | $E_g$ | | |
|---|---|---|---|---|---|---|---|---|---|---|
| | **Model** | $R$ | **MAE** | $N_{\varepsilon>1}$ | $R$ | **MAE** | $N_{\varepsilon>1}$ | $R$ | **MAE** | $N_{\varepsilon>1}$ |
| **Elemental** | **RFR** | 0.93 | 0.41 | 69 (7.9%) | 0.92 | 0.41 | 64 (7.3%) | 0.80 | 0.19 | 23 (2.6%) |
| | **GBR** | 0.93 | 0.41 | 62 (7.1%) | 0.92 | 0.41 | 68 (7.8%) | 0.70 | 0.28 | 29 (3.3%) |
| | **KRR** | 0.90 | 0.50 | 100 (11.5%) | 0.88 | 0.51 | 111 (12.7%) | 0.79 | 0.22 | 22 (2.5%) |
| | **SVR** | 0.87 | 0.62 | 137 (15.7%) | 0.84 | 0.61 | 142 (16.3%) | 0.73 | 0.25 | 31 (3.6%) |
| | **MLPR** | 0.90 | 0.49 | 104 (11.9%) | 0.88 | 0.50 | 125 (14.3%) | 0.73 | 0.26 | 27 (3.1%) |
| **DOS** | **RFR** | 0.99 | 0.18 | 6 (0.7%) | 0.98 | 0.18 | 5 (0.6%) | 0.88 | 0.15 | 10 (1.1%) |
| | **GBR** | 0.99 | 0.16 | 7 (0.8%) | 0.99 | 0.16 | 4 (0.5%) | 0.90 | 0.15 | 7 (0.8%) |
| | **KRR** | 0.999 | 0.19 | 6 (0.7%) | 0.98 | 0.19 | 6 (0.7%) | 0.86 | 0.19 | 10 (1.1%) |
| | **SVR** | 0.96 | 0.32 | 16 (1.8%) | 0.95 | 0.35 | 24 (2.8%) | 0.69 | 0.31 | 17 (1.9%) |
| | **MLPR** | 0.96 | 0.34 | 43 (4.9%) | 0.95 | 0.33 | 36 (4.1%) | 0.76 | 0.24 | 26 (3.0%) |

**Figure S3.** Correlation plots between the true PBE0 and the ML predicted bandgap edges (VBM or CBM) using the full database, for each studied model. All values are given in eV. The top two rows show the results using only elemental features, while the bottom two rows also include DOS features. In blue and green are represented the train and test data, respectively. *R* and MAE values are provided in the insets.

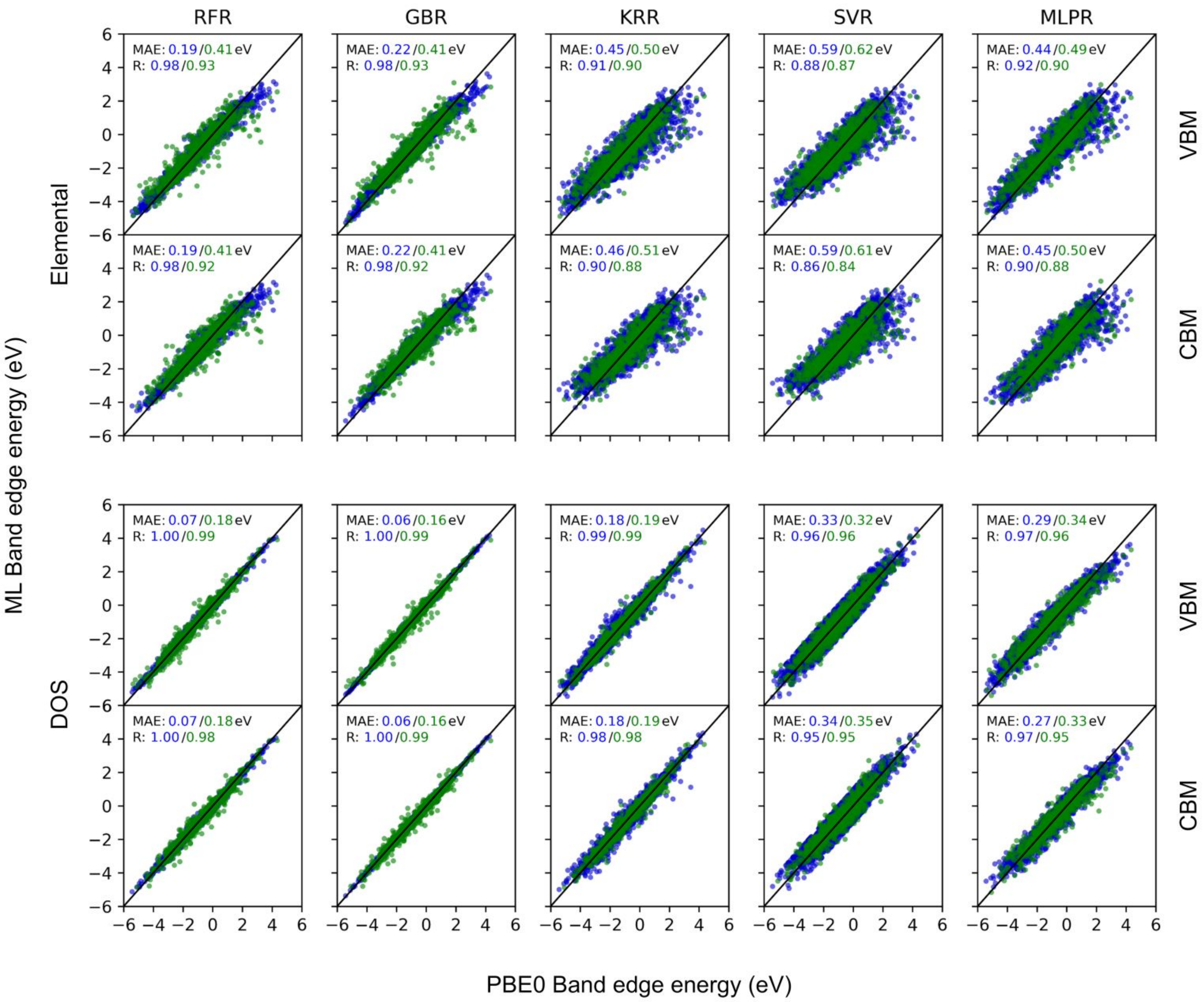

**Figure S4.** Correlation plots between the true PBE0 and the ML predicted bandgap extracted from the band edge prediction, for each studied model. The top row shows the results using only elemental features, while the bottom row also includes DOS features. In blue and green are represented the training and test datasets, respectively. *R* and MAE (in eV) values are shown in each panel.

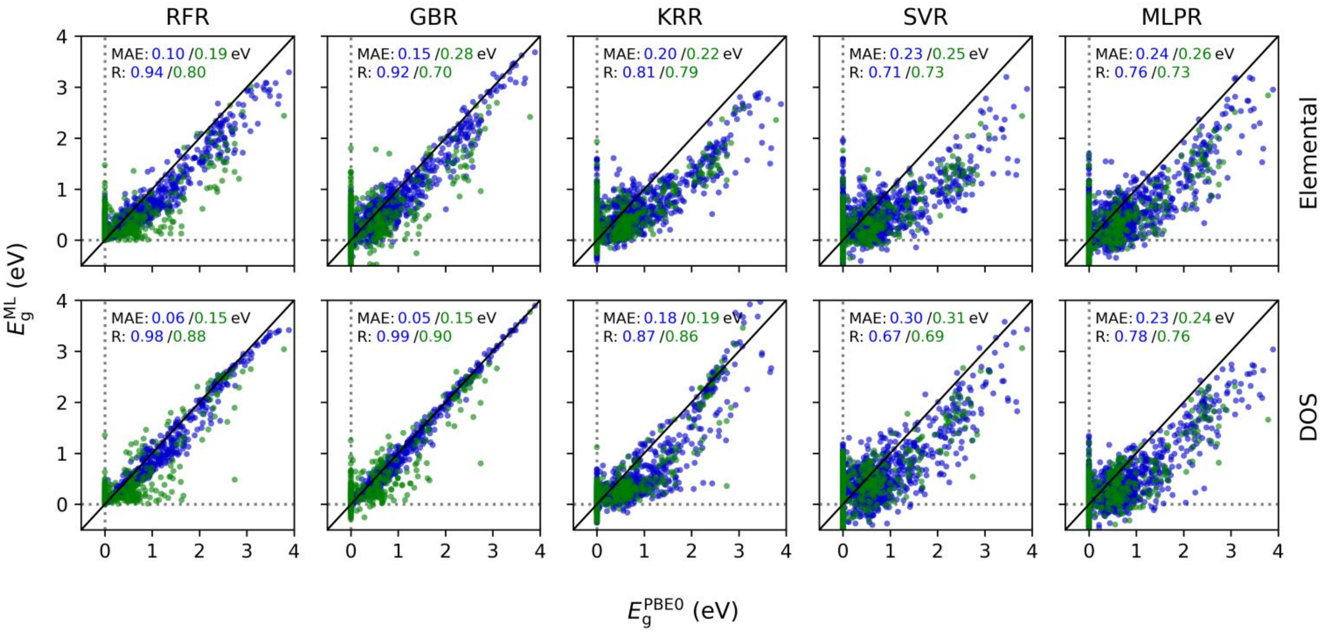

**Table S4**. Evaluation metrics for the classification of MXenes into metallic or semiconductor, for all studied models, including gradient boosting classifier (GBC), random forest classifier (RFC), support vector classifier (SVC), multi-layer perceptron classifier (MLPC), and logistic regression (LR). The accuracy, precision, recall, true predicted *vs.* false negative ratio and area under receiver operating characteristic (ROC) curve (AUC) are given for two independently-trained models, one with only elemental features and another that also includes DOS features.

| | **Elemental** | | | | **DOS** | | | |
|---|---|---|---|---|---|---|---|---|
| **Model** | **Accuracy** | **Precision** | **Recall** | **AUC** | **Accuracy** | **Precision** | **Recall** | **AUC** |
| **RFC** | 0.86 | 0.79 | 0.55 | 0.90 | 0.91 | 0.91 | 0.64 | 0.97 |
| **GBC** | 0.85 | 0.75 | 0.47 | 0.89 | 0.92 | 0.86 | 0.73 | 0.95 |
| **LR** | 0.83 | 0.69 | 0.44 | 0.81 | 0.89 | 0.83 | 0.63 | 0.92 |
| **SVC** | 0.86 | 0.72 | 0.67 | 0.91 | 0.83 | 0.60 | 0.59 | 0.84 |
| **MLPC** | 0.81 | 0.64 | 0.38 | 0.82 | 0.87 | 0.71 | 0.66 | 0.92 |

**Figure S5.** Confusion matrices for the true PBE0 and ML predicted classification of MXenes into metallic (m) or semiconductor (s), for the studied models. The top row shows the results using only elemental features, while the bottom row also includes DOS features.

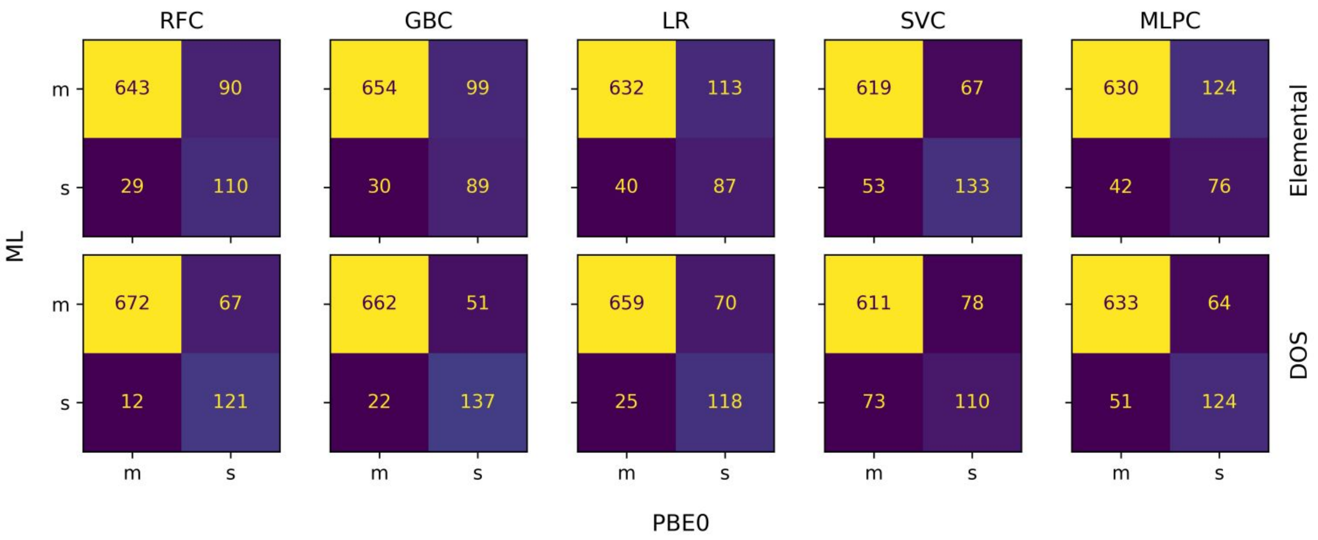

**Figure S6.** ROC curve for the classification of MXenes between metallic and semiconductors, showing the true positive rate (TPR) with respect the false positive rate (FPR). In green is filled the AUC, with its corresponding value on the corner. The diagonal black dashed line corresponds to a random classification (AUC = 0.5). The top row shows the results using only elemental features, while the bottom row also includes DOS features.

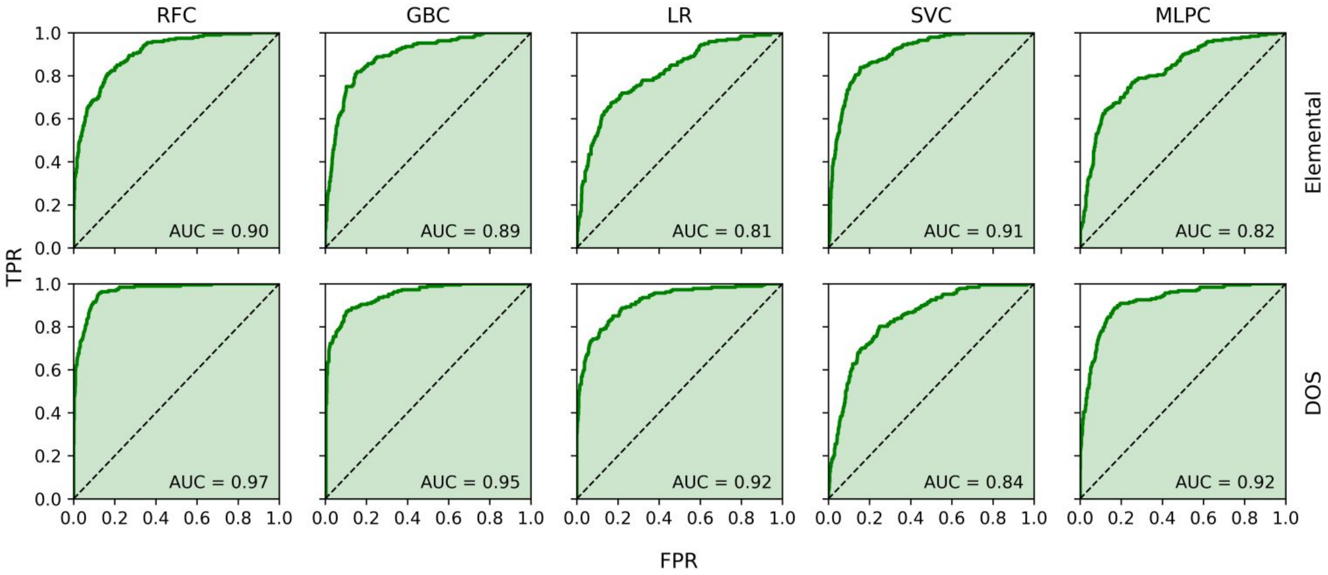

**Table S5.** Evaluation metrics for the different models using only semiconductor MXenes. The *R*, MAE (in eV), and and $N_{\varepsilon>1}$ are displayed for two independently-trained models, one with only elemental features and another that also includes DOS features. The $N_{\varepsilon>1}$ also includes the percent of cases it corresponds, between parentheses.

| | **Elemental** | | | **DOS** | | |
|---|---|---|---|---|---|---|
| **Model** | ***R*** | **MAE / eV** | $N_{\varepsilon>1}$ | ***R*** | **MAE / eV** | $N_{\varepsilon>1}$ |
| **RFR** | 0.89 | 0.28 | 4 (2.1%) | 0.92 | 0.24 | 1 (0.5%) |
| **GBR** | 0.83 | 0.34 | 7 (3.7%) | 0.92 | 0.24 | 2 (1.1%) |
| **KRR** | 0.91 | 0.25 | 2 (1.1%) | 0.90 | 0.27 | 2 (1.1%) |
| **SVR** | 0.89 | 0.29 | 2 (1.1%) | 0.88 | 0.31 | 4 (2.1%) |
| **MLPR** | 0.89 | 0.28 | 3 (1.6%) | 0.85 | 0.31 | 5 (2.6%) |

**Figure S7.** Correlation plots between the true PBE0 and the ML predicted bandgap for semiconductor MXenes, for each studied model. The top row shows the results using only elemental features, while the bottom row also includes DOS features. In blue and green are represented the training and test data sets, respectively. *R* and MAE (in eV) values are shown in each panel.

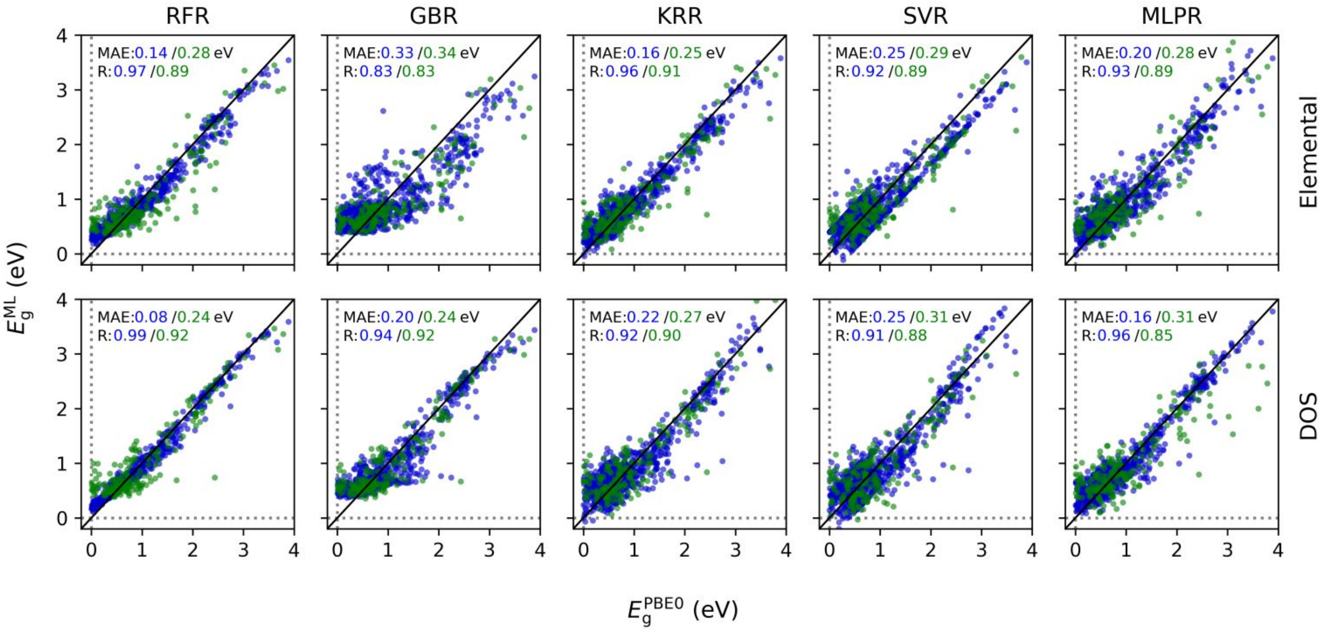

**Figure S8.** Learning curves for the bandgap using the best-performing model (GBC+RFR). In blue and green are represented the averaged MAE (in eV) for the train and test sets, respectively. The shaded regions around the curves represent the standard deviation in the MAE across 10 different random splits.

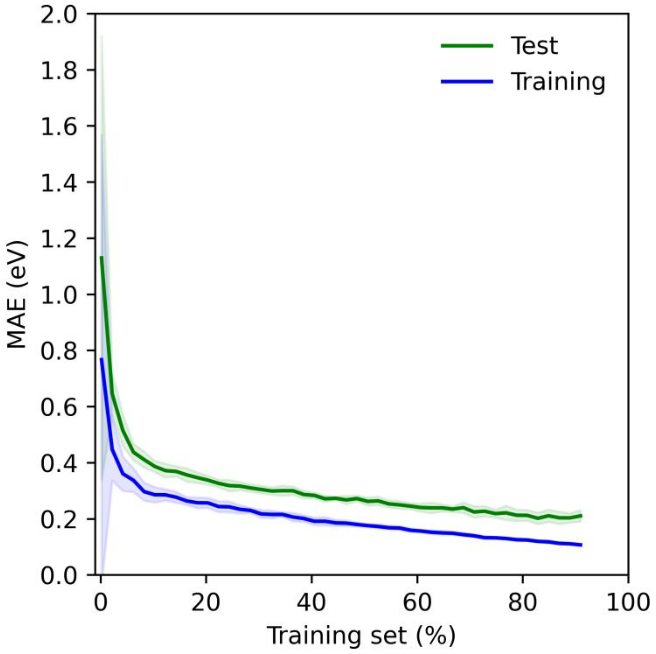

**Figure S9.** Linear correlations between PBE and PBE0 bandgap values for different terminated MXenes. This analysis includes only cases where a PBE bandgap is present. For each termination, the correlation coefficient *R* is provided (shown color-coded), along with the overall *R* value considering all cases collectively (solid black line).

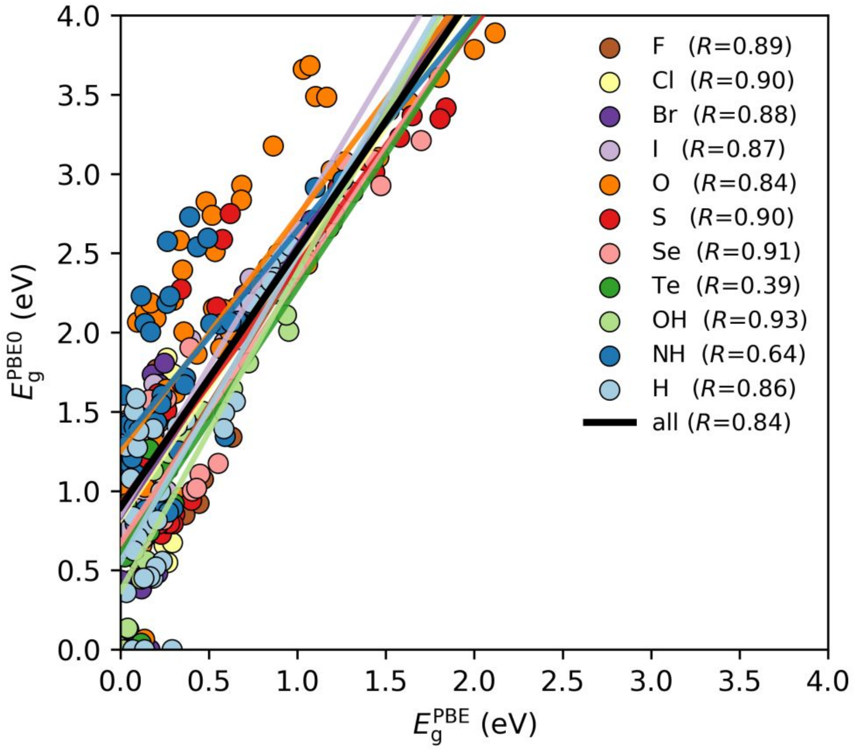

**Figures S10.** Analysis of the chemical space using the *t*-distributed stochastic neighbour embedding (*t*-SNE) projection. This algorithm reduces high-dimensional feature space into a 2D representation, where each point corresponds to a system in the database. The proximity between points reflects the chemical similarity of the corresponding MXene structures. In green, red, and purple are marked the Sc-, Y-, and La-MXenes, while in gray is the rest of the systems. A perplexity value of 20 is used, which determines the number of nearest neighbors considered when preserving local structure in the projection.

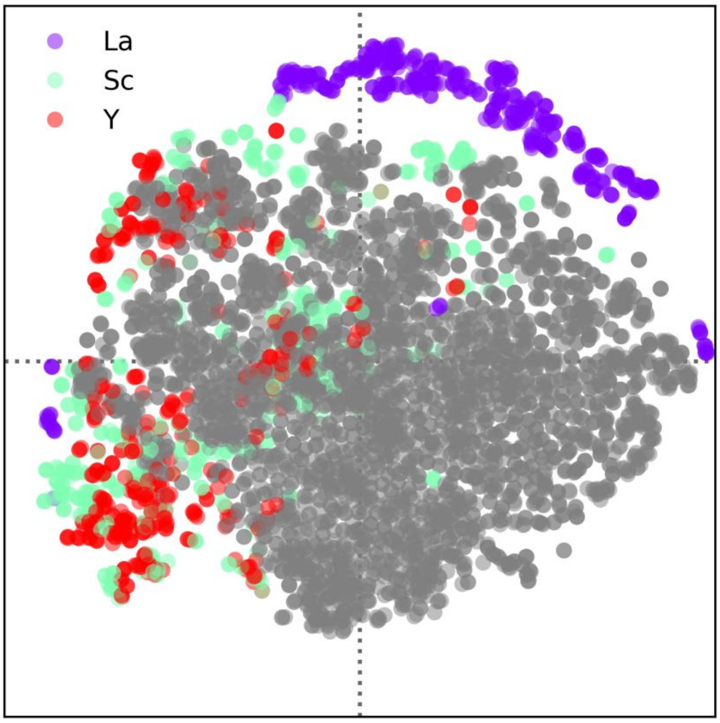

**Table S6.** Validation results for the best model (GBC+RFR) for the 14 optimal and 14 random selected cases. The ML predicted gap, $E_g^{ML}$, the PBE0 gap, $E_g^{PBE0}$, and absolute gap difference, $\Delta E_g$, are given in eV. In the last two columns are also included the predictions using the GBC coupled with GBR, $E_g^{GBR}$, or KRR, $E_g^{KRR}$, regressors.

| type | MXene | stack | hollow | $E_g^{ML}$ | $E_g^{PBE0}$ | $\Delta E_g$ | $E_g^{GBR}$ | $E_g^{KRR}$ |
|---|---|---|---|---|---|---|---|---|
| optimal | $La_2CF_2$ | ABC | $H_M$ | 2.30 | 2.37 | 0.07 | 2.16 | 2.25 |
| optimal | $La_2CCl_2$ | ABC | $H_M$ | 1.96 | 2.02 | 0.06 | 1.80 | 1.27 |
| optimal | $La_2CBr_2$ | ABC | $H_M$ | 1.93 | 1.92 | 0.01 | 1.80 | 1.11 |
| optimal | $La_2CI_2$ | ABC | $H_M$ | 1.84 | 1.79 | 0.05 | 1.50 | 1.13 |
| optimal | $La_2CO_2$ | ABC | $H_{MX}$ | 1.23 | 2.04 | 0.81 | 1.10 | 0.46 |
| optimal | $La_2CS_2$ | ABC | $H_{MX}$ | 2.45 | 2.48 | 0.03 | 2.35 | 1.75 |
| optimal | $La_2NS_2$ | ABC | $H_{MX}$ | 2.42 | 2.79 | 0.37 | 2.52 | 2.27 |
| optimal | $La_2CSe_2$ | ABC | $H_{MX}$ | 2.74 | 2.71 | 0.03 | 2.46 | 2.77 |
| optimal | $La_2CTe_2$ | ABC | $H_{MX}$ | 1.33 | 2.61 | 1.28 | 0.85 | 0.72 |
| optimal | $La_2CH_2$ | ABC | $H_M$ | 1.88 | 1.81 | 0.07 | 1.87 | 1.29 |
| optimal | $La_2C(OH)_2$ | ABC | $H_M$ | 1.88 | 1.72 | 0.16 | 1.83 | 1.57 |
| optimal | $La_3N_2S_2$ | ABC | $H_{MX}$ | 2.06 | 2.22 | 0.16 | 2.01 | 0.86 |
| optimal | $La_3C_2(NH)_2$ | ABC | $H_M$ | 1.60 | 0.73 | 0.87 | 1.29 | 0.84 |
| optimal | $La_4N_3S_2$ | ABC | $H_{MX}$ | 1.76 | 1.74 | 0.02 | 1.71 | 0.92 |
| random | $La_2CF_2$ | ABA | H | 2.25 | 2.35 | 0.10 | 2.17 | 1.93 |
| random | $La_2NI_2$ | ABC | $H_{MX}$ | 0.84 | 0.28 | 0.56 | 0.65 | -0.25 |
| random | $La_2NO_2$ | ABC | $H_M$ | 0 | 0 | 0 | 0 | 0 |
| random | $La_2NTe_2$ | ABA | $H_{MX}$ | 0 | 0 | 0 | 0 | 0 |
| random | $La_2N(NH)_2$ | ABC | $H_M$ | 1.05 | 1.21 | 0.16 | 0.94 | 0.25 |
| random | $La_3C_2Cl_2$ | ABC | $H_{MX}$ | 0.55 | 0.68 | 0.13 | 0.46 | 0.32 |
| random | $La_3C_2I_2$ | ABA | $H_X$ | 0 | 0.55 | 0.55 | 0.00 | 0.00 |
| random | $La_3N_2O_2$ | ABA | $H_X$ | 1.90 | 2.11 | 0.21 | 1.88 | 1.47 |
| random | $La_3C_2(OH)_2$ | ABC | $H_X$ | 0 | 0 | 0 | 0 | 0 |
| random | $La_4C_3I_2$ | ABA | $H_X$ | 0 | 0.43 | 0.43 | 0 | 0 |
| random | $La_4C_3S_2$ | ABA | $H_{MX}$ | 0.76 | 0 | 0.76 | 0.56 | -0.13 |
| random | $La_4N_3Se_2$ | ABC | $H_{MX}$ | 0 | 0 | 0 | 0 | 0 |
| random | $La_4C_3Te_2$ | ABC | $H_X$ | 1.42 | 1.39 | 0.03 | 1.18 | 0.05 |
| random | $La_4C_3H_2$ | ABA | H | 0 | 0 | 0 | 0 | 0 |

**Figure S11.** Band alignment diagrams relative to the $H^+/H_2$ and $H_2O/O_2$ redox potential energy levels, for the other eight cases with non-suitable band alignment identified through the screening. The blue and orange bars indicate the valence band (VB) and conduction band (CB), respectively. For the Janus chalcogen-terminated MXenes (right), the band edge position is represented for each surface ($H_M$ or $H_X$). The $La_2NS_2$ case, although being a Janus $H_{MX}$ structure, presents a non-noticeable difference between the band alignment of each surface. In black and gray dashed lines is represented the redox potentials at $pH$ = 0 or $pH$ = 7, respectively.

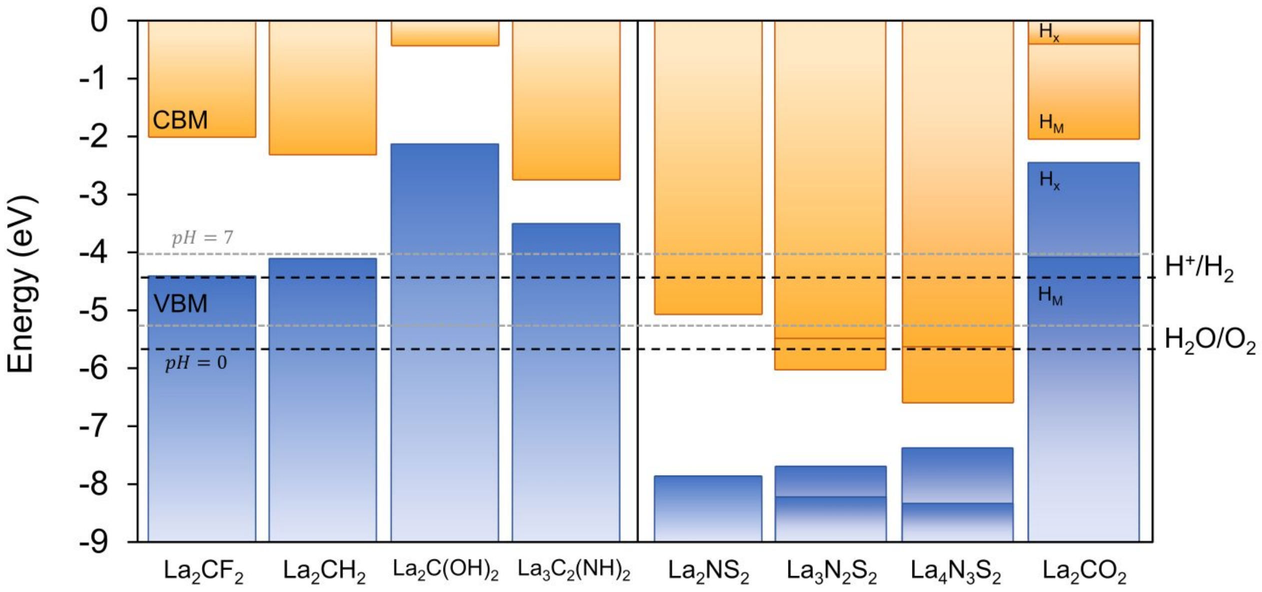

**References**

(1) Pedregosa, F.; Varoquaux, G.; Gramfort, A.; Michel, V.; Thirion, B.; Grisel, O.; Blondel, M.; Prettenhofer, P.; Weiss, R.; Dubourg, V.; Vanderplas, J.; Passos, A.; Cournapeau, D.; Brucher, M.; Matthieu, P.; Duchesnay, É. Scikit-learn: Machine learning in Python. *J. Mach. Learn. Res.* **2011**, *12*, 2825–2830.
